# Functional Interactions Between the Parafascicular Thalamic Nucleus and Motor Cortex Are Altered in Hemiparkinsonian Rat

**DOI:** 10.3389/fnagi.2022.800159

**Published:** 2022-05-23

**Authors:** Min Li, Xiao Zhang, Qin He, Dadian Chen, Feiyu Chen, Xiaojun Wang, Shuang Sun, Yue Sun, Yuchuan Li, Zhiwei Zhu, Heyi Fang, Xiaoman Shi, Xiaomeng Yao, Haiji Sun, Min Wang

**Affiliations:** ^1^Key Laboratory of Animal Resistance Biology of Shandong Province, College of Life Science, Shandong Normal University, Jinan, China; ^2^Editorial Department of Journal of Shandong Jianzhu University, Jinan, China; ^3^School of International Education, Qilu University of Technology, Jinan, China; ^4^The First Hospital Affiliated With Shandong First Medical University, Jinan, China; ^5^School of Nursing, Qilu Institute of Technology, Jinan, China

**Keywords:** local field potential (LFP), Parkinson’s disease (PD), parafascicular thalamic nucleus, motor cortex, spike, coherence, rat

## Abstract

Parkinson’s disease (PD) is characterized by aberrant discharge patterns and exaggerated oscillatory activity within basal ganglia-thalamocortical circuits. We have previously observed substantial alterations in spike and local field potential (LFP) activities recorded in the thalamic parafascicular nucleus (PF) and motor cortex (M1), respectively, of hemiparkinsonian rats during rest or catching movements. This study explored whether the mutual effects of the PF and M1 depended on the amplitude and phase relationship in their identified neuron spikes or group rhythmic activities. Microwire electrode arrays were paired and implanted in the PF and M1 of rats with unilateral dopaminergic cell lesions. The results showed that the identified PF neurons exhibited aberrant cell type-selective firing rates and preferential and excessive phase-locked firing to cortical LFP oscillations mainly at 12–35 Hz (beta frequencies), consistent with the observation of identified M1 neurons with ongoing PF LFP oscillations. Experimental evidence also showed a decrease in phase-locking at 0.7–12 Hz and 35–70 Hz in the PF and M1 circuits in the hemiparkinsonian rats. Furthermore, anatomical evidence was provided for the existence of afferent and efferent bidirectional reciprocal connectivity pathways between the PF and M1 using an anterograde and retrograde neuroanatomical tracing virus. Collectively, our results suggested that multiple alterations may be present in regional anatomical and functional modes with which the PF and M1 interact, and that parkinsonism-associated changes in PF integrate M1 activity in a manner that varies with frequency, behavioral state, and integrity of the dopaminergic system.

## Introduction

Much of the motor impairment associated with Parkinson’s disease (PD) is presumed to arise from aberrant frequency-specific oscillatory and/or neuronal single-unit activity across the cortico-basal ganglia-thalamocortical circuit (Wang et al., 2015; West et al., 2018; Cagnan et al., 2019; Holt et al., 2019; Underwood and Parr-Brownlie, 2021). Profound aberrant discharge patterns and presence of exaggerated oscillatory activity across the basal ganglia (Avila et al., 2010; Brazhnik et al., 2012, 2014; Halje et al., 2012; Quiroga-Varela et al., 2013; Devergnas et al., 2014; Rossant et al., 2016), thalamic nuclei (Parr-Brownlie et al., 2009; Bosch-Bouju et al., 2014), and motor cortex (Parr-Brownlie et al., 2007; Pasquereau and Turner, 2013; Pasquereau et al., 2015; Hyland et al., 2019) have been observed. Dopamine depletion in PD triggers pathologic alterations not only among individual brain sites but also throughout the cortico-basal ganglia-thalamocortical circuit. These interactions are mediated by the prototype of anatomical connections and by rapidly and selectively engaging functional neuronal connections (Jin et al., 2016; Zhang et al., 2019a; Gaidica et al., 2020). Therefore, a significant challenge in modern neuroscience is determining alterations in functional connectivity in the cortico-basal ganglia-thalamocortical circuit.

Among the components of the cortico-basal ganglia-thalamocortical circuit, the thalamus is a central hub in nearly all motor, sensory, and associative circuits and is, therefore, well-positioned to regulate circuit-wide neuronal activities (Sherman, 2016; Gaidica et al., 2018, 2020; Halassa and Sherman, 2019). Therefore, altered thalamic activity has been assumed to underlie the pathogenesis of motor deficits in individuals with PD (Bosch-Bouju et al., 2014; Brazhnik et al., 2016; Cagnan et al., 2019). Among many thalamic nuclei, the thalamic intralaminar nuclei are part of the higher-order thalamus, which receives little sensory input and, instead, forms extensive cortico-basal ganglia-thalamocortical circuits, a key node in this circuit that controls movement (Saalmann, 2014). As a component of the intralaminar nuclei, the thalamic centromedian–parafascicular (CM/PF) complex, mainly represented by the parafascicular nucleus (PF) in rodents, differs from other intralaminar thalamic groups in terms of its rich connectivity with the basal ganglia (Smith et al., 2004, 2014). Evidence from postmortem human brain studies indicates that the CM/PF complex presents 30–40% cell loss in individuals with PD, unlike other thalamic nuclei that maintain their integrity throughout the disease (Henderson et al., 2000a,b; Halliday, 2009). A similar pattern of degeneration was observed in the CM/PF complex of parkinsonian monkeys (Villalba et al., 2014). Altered PF activity in rodents has been assumed to underlie the pathogenesis of PD-related motor deficits (Ni et al., 2000; Parr-Brownlie et al., 2009; Smith et al., 2014; Wang et al., 2016). Furthermore, after dopamine depletion, thalamostriatal synapses are a site of maladaptive changes in mice with PD (Parker et al., 2016). A recent study implies that PF innervation of the striatum induces alterations in thalamostriatal network activity related to PD pathophysiology (Tanimura et al., 2019). Two populations of PF neurons were previously identified whose activity correlated with distinct aspects during rest and movement, and 6-OHDA induced modifications in PF spike and local field potential (LFP) activities in hemiparkinsonian rats (Wang et al., 2016). In addition, the two populations of PF neurons were associated with different levels of increased spike-LFP synchronization with the dorsal striatum in the hemiparkinsonian rats (Zhang et al., 2019b).

Numerous primate and rodent studies have shown that CM/PF or PF neurons consist of large anatomically and physiologically distinct neuronal populations that extend topographically organized projections to the striatum and provide relatively minor anatomically collaterals to and from the cerebral cortex (Sadikot and Rymar, 2009; Mandelbaum et al., 2019; Stayte et al., 2021). The PF is well-positioned to reciprocally modulate information flow between the cerebral cortex and the thalamus to a certain extent (Sherman, 2017; Halassa and Sherman, 2019). However, the mechanisms by which different groups of PF neurons may regulate information transmission in cortical circuits remain unknown. Therefore, understanding the neural interaction mechanism between the PF and M1 in different groups of neurons is challenging. Recently, our research group discovered that neurons in the primary motor cortex (M1) of dopaminergic-lesioned rats were accompanied by decreased spike firing rate, reshaping the firing pattern and aberrant LFP-specific frequency oscillations (Li et al., 2021). Motivated by these studies, we hypothesize that the PF may modulate cortical processes to regulate information flow and that the functional relevance of the interaction of the PF with M1 would be involved in the pathophysiology of PD or its animal models. We used multielectrode arrays to record spikes and LFPs simultaneously from the PF and M1 *in vivo* of animals at rest and performed behavioral tasks to define how chronic depletion of dopamine, which occurs in patients with PD, alters the temporal organization of electrical activity, and to infer changes in the neural transmission that occur in PF-M1 circuits following chronic dopamine depletion.

## Materials and Methods

### Animals and Behavioral Training

Male Wistar rats weighing 280–320 g (Shandong University, China) were housed to a cage under environmentally controlled conditions and 14-h10-h light/dark cycles, with free access to water and limited food. All experimental procedures were conducted in accordance with the National Institute of Health Guide for the Care and Use of Laboratory Animals (NIH Publication No. 8023, revised 1978) and approved by the Shandong Normal University Ethics Review Board (Protocol Number: AEECSDNU2020016).

**FIGURE 1:**
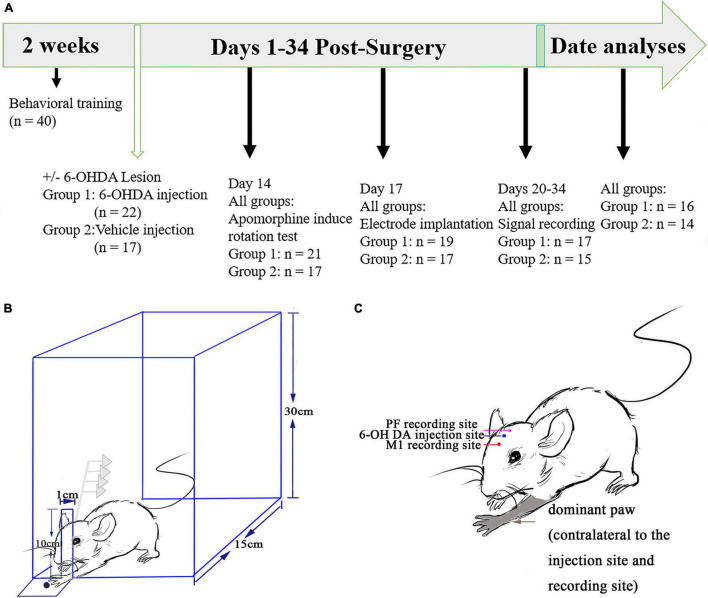
Experimental design and timeline. **(A)** Prior to and following surgery to induce a unilateral 6-hydroxydopamine (6-OHDA) lesion of the medial forebrain bundle, all rats (*n* = 40) underwent behavioral training and were monitored to execute the catching task to obtain food through a slit using their preferred forepaws. All the rats performed an apomorphine-induced rotation test 2 weeks after the surgery. A multiunit recording electrode was implanted into the primary motor cortex (M1) and thalamic parafascicular nucleus (PF) of all the lesioned and control rats 2 weeks after the first surgery. Signal recording began from 20 to 34 days after surgery for further data analyses. **(B)** Schematic diagram of the multiunit recordings from the M1 and PF nucleus. Slits in the wall of the test chamber were placed individually at the contralateral side of the preferred forepaws. This experiment provides an example indicating that a rat could catch food through a slot with its left forepaw. **(C)** Illustration showing a rat whose left forepaw dominates after behavioral training. Recording electrodes (M1 and PF) and intracerebral injection of 6-OHDA (or vehicle in the control group) are contralateral to the preferred forepaw.

The specific experimental procedures are shown in Figure 1A. No statistical method was used to determine sample size. The sample size was arbitrarily set to 40 (two groups). One week before surgery, all the rats (*n* = 40) were trained to execute reach-to-grasp food movements, i.e., to retrieve a food pellet with their forepaw, and the dominant forepaw was determined using a previously described method (Wang et al., 2016; Li et al., 2021). Briefly, the animals reached through a small slit (1 cm) in the wall of a transparent plastic test chamber (homemade, 20 cm × 15 cm × 30 cm) to obtain food pellets (grinding standard rat chow, KeAoXie Li Company, Beijing, China) placed on a tray using their preferred forepaws (Figure 1B). The tray was mounted in front of the slit outside the test chamber. Reach-to-grasp food movements were detected by interruption of a light beam generated with an infrared obstacle avoidance sensor (E18-D80NK) connected to the OmniPlex D System *via* digital input–output interfacing. In parallel with both behavioral and screen recorders, CamStudio (MicroImages, Inc., United States) was used to record continuous real-time behaviors with synchronization of electrophysiological recordings through computer interaction. The rats received daily sessions consisting of 90 trials, separated into three blocks of 30 trials. One trial began when an animal approached the slit and grabbed the food and ended when it retracted the preferred forepaws. The entire session required 30 min to complete, with an hour break between every session, and the animals were trained 7 days per week. Behavioral experiments were performed between 9 and 12 am.

### Unilateral Lesion of the Nigrostriatal Pathway

After the behavioral training period, the rats were randomly divided into two groups. One group [6-hydroxydopamine (6-OHDA)-treated group, *n* = 22 rats] received a unilateral injection of 6-OHDA in the medial forebrain bundle (MFB, contralateral to the preferred paw) (Figure 1C). The other group of rats (control group, *n* = 17 rats) received a unilateral injection of vehicle (0.02% ascorbic acid in physiological saline) in the medial forebrain bundle (MFB, contralateral to the preferred paw). The protocol used to induce the destruction of the nigrostriatal pathway was applied as previously described (Brazhnik et al., 2012). Thirty minutes prior to intracerebral injections of 6-OHDA into the MFB, desipramine hydrochloride [15 mg/kg, i.p.; Sigma, catalog# 58-28-6 (year 2019)] was administered to protect noradrenergic neurons. The animals were anesthetized with urethane [1 g/kg i.p., Sigma, catalog#51-79-6 (year 2019)]. A hole was drilled in the skull at –2.16 mm posterior to the bregma and 2.1 mm lateral to the sagittal suture contralateral to the preferred paw determined *via* behavioral training. Two micrograms per microliter of 6-OHDA in 3 μl [0.9% saline with 0.02% ascorbic acid, Sigma, catalog #28094-15-7 (year 2019)] was injected with a microsyringe into the MFB (8.5 mm ventral to the skull surface) at a rate of 0.5 μl/min for over 6 min. The needle of the microsyringe remained in the injection site for another 6–10 min to prevent neurotoxin diffusion. The control rats only received the same volume of vehicle at the same coordinates. Then, the skull incision was sutured, and the long-acting analgesic carprofen [5 mg/kg, Sigma, catalog #53716-49-7 (year 2019)] was injected subcutaneously. The rats received postoperative care and were monitored for 1 week after surgery.

The efficacy of dopaminergic lesions was assessed 7 days after the surgery by treatment with apomorphine [0.05 mg/kg s.c., Sigma, catalog# A4393 (year 2019)]. The animals that performed more than 210 rotations contralateral to the lesioned site within 30 min were confirmed to have a successful lesion (Yuan et al., 2004; Geng et al., 2016a,b). Only these animals were used as the 6-OHDA-treated group for behavioral tests and electrophysiological recordings. The degree of dopaminergic neuron degeneration was assessed in postmortem tissues by immunohistochemical staining for tyrosine hydroxylase (TH).

### Recording Electrode Implantation

Each rat was initially anesthetized with urethane (1 g/kg i.p.; Sigma, St. Louis, MO, United States) and mounted in a stereotaxic frame. Holes were drilled in the skull over the target recording sites contralateral to the hemispheres for the preferred paw. An electrode array constructed from two bundles of 8 nickel–chromium and HFV natural-insulated microwires (12.5 μm in diameter; California Fine Wire, Grover Beach, CA, United States) plus an additional seventeenth wire with uninsulated silver wires (125 μm) as ground wires and a nickel–chromium wire (125 μm) as a reference were used to monitor and decrease background noise, especially during animal forelimb catching periods. One bundle of 8 independent microwires (2 × 4 pattern, with two rows with an interval of 100 μm) of the electrode array was implanted on the skull above the target coordinates for the PF (–4.2 ± 0.1 mm posterior to the bregma, 1.2 ± 0.1 mm lateral to the sagittal suture, and 6 ± 0.5 mm ventral to the skull surface), and another bundle of 8 independent microwires was implanted to detect M1 layer 5 (+1 ± 0.2 mm anterior to the bregma, 2 ± 0.2 mm lateral to the sagittal suture, and 1.9 ± 0.1 mm ventral to the skull surface), which pertain to the hemisphere ipsilateral to the 6-OHDA infusion (Figure 1C, according to the atlas of Paxinos and Watson, 2007). Four stainless steel screws were implanted onto the skull as anchors and connected with an uninsulated silver wire serving as electric ground. The skull was then sealed with a layer of agar, and then the microwire arrays and stainless-steel screws were affixed onto the skull with dental cement. Ultimately, the animals received the same long-acting analgesic and postoperative care as described above after lesioning of the nigrostriatal pathway.

### Electrophysiological Data Acquisition and Selection

All extracellular recordings were performed 1 week post-surgery and were conducted weekly for 5–8 weeks. Extracellular spike trains and LFPs were simultaneously recorded during epochs of inattentive rest and forelimb reaching movements. During the recording sessions, spike trains and LFPs were amplified and filtered using Plexon systems (OmniPlex D Neural Data Acquisition, Inc., Dallas, TX, United States). Spikes were amplified (10,000×) by bandpass filtering (0.3–3 kHz) and sampled at 20 kHz. LFPs were amplified (1000×) and bandpass filtered (0.7–200 Hz). A notch filter was used to eliminate line noise at 50 Hz. Sampling rates were 40 kHz for spikes and 1 kHz for LFPs. Discriminated spike and LFP signals were digitized and stored in individual text files for subsequent offline analysis.

For evaluations of neuronal activity, epochs of extracellular unit activity and simultaneous LFP recordings free of major artifacts were used in the analysis. Direct observation and videotaped motor behaviors were used to select periods representing different behavioral states. All the recorded information was further assessed using Offline Sorter version 4 (Plexon, Inc., Dallas, TX, United States), NeuroExplorer version 5 (NEX Technologies, Littleton, MA, United States), and MATLAB (MathWorks, Natick, MA, United States) scripts. Each recording session for all the rats included periods of rest and forelimb catching movement, as previously described (Wang et al., 2016). By direct visual observation and video recording, epochs representing different behavioral states were selected. Epochs of the remaining periods were categorized as inattentive rest states, i.e., when the rats were awake but not attentive to their tasks (when the rats were awake and maintained the same posture). Care was taken to prevent surrounding distractions that would alert them. The analyses were focused on self-initiated, spontaneous catching movements to examine movement-related neuronal activities. These movements were defined as those involving forelimb movements (determined from video recordings). Movement periods were determined using a combination of videos of behavior (30 frames/s) and the Infrared Obstacle Avoidance Sensor (E18-D80NK). The analyses were focused on perievent raster plots and perievent time histograms (PETHs) that revolved around catching movement-related neuronal activities. PETHs (40 ms bins) were smoothed with a sliding seven-point Gaussian window using NeuroExplorer version 5 routines (NEX Technologies, Littleton, MA, United States). Only neurons recorded during the execution of such movement periods were considered for further analysis of movement-related neuronal activities.

### Electrophysiological Data Analysis

During the experiments and analysis, the investigators were blinded to the experimental groups. Animals were identified by earmarks that assigned numbers to each, and investigators were announced only after completing the analysis.

#### Identification and Classification of Neurons

Based on recorded continuous voltage traces of the electrode array, individual neuron action potentials were detected and sorted using the offline sorter version 4 software^1^, and then the data were manually checked to ensure that the separated cluster boundaries were clear and that inconformity spike waveforms were rejected. Putative spike activity was isolated with previously described spike sorting procedures (Nicolelis et al., 2003; Isomura et al., 2009; Kaufman et al., 2013; Saiki et al., 2014; Wang et al., 2016; Li et al., 2021). The first step was to set a voltage threshold for each of the channel processing signals. The threshold value was sufficiently higher than the noise level, in which some obvious artifacts in the signals were removed manually if a spike did not exhibit a high quality of separation from the background noise or did not match with sorted neurons. Then, the selected single units were separated based on several waveform parameters and distinguishing features in the offline sorter. Initially, a principal component (PC) analysis was conducted using all unsorted spike waveforms. Different types of neurons were automatically isolated from different clusters displayed with each point in the 2D/3D PC space using the K-means clustering algorithm. The clusters were further inspected, and units that were not easily identified automatically were manually added or deleted. The quality metrics of the separate clusters were calculated by determining isolation distance (Iso Dist), and LRatio measures included the parametric statistic of multivariate analysis of variance. Iso Dist is a measure of how distant non-unit spikes are from spikes in the unit under consideration; thus, a large Iso Dist value indicates a well-separated unit. LRatio is a measure of the amount of noise near a unit; thus, a low value of L-ratio indicates that a unit is well separated (Schmitzer-Torbert et al., 2005). Next, by selecting a group of points from the PC analysis, interspike interval (ISI) histograms were calculated, in which each unit exhibited a clearly recognizable refractory period (>2 ms) and had a characteristic and distinct ISI shape. Finally, spike duration was the trough-to-peak measure (between the first negative deflection and the peak of the second positive deflection of a spike waveform), which was easily detectable and provided the most reliable separation of two classes (Barthó et al., 2004). Generally, for each of these data segments, single units were discriminated based on multiple parameters: waveform voltage and shape, 2D/3D PC clusters, ISIs, and cluster separation statistics calculated for each recorded channel. All allowed excellent separations of waveforms collected from single or multiple electrodes into distinct categories.

#### Spectral Analysis of Local Field Potentials

LFP activity was analyzed by computing spectral power distributions using Chronux 2.12^2^ and the in-house MATLAB software (MathWorks, Natick, MA, United States). LFP was calculated using the multiple tapers method (time band with product = 5, number of tapers = 9) with a window size of 0.5 s, time step of 0.1 s, and sampling frequency of 1,000 applied separately to each window. Similar to previous studies, relevant LFP power spectra were analyzed by focusing on four main bands.7–12, 12–35, 35–70, 70–100 Hz, and 100–200 Hz to examine frequency band-specific characteristics (Rossant et al., 2016). The power for each band was computed as a function to provide a ratio across the power for an entire signal (0.7–200 Hz), which was reported as the ratio of the power for a relevant band to total power to overcome effects attributed to individual non-specific differences in absolute power.

### Analysis of the Relationship Between Spikes and Local Field Potentials

#### Coherence Value

The relationship between spikes and LFPs after 6-OHDA treatment was defined as the primary endpoint of this study. Functional relationships between M1 and PF were estimated by calculating coherence. Coherence provides a normalized measure of the linear correlation between signals in the frequency domain. Coherence between two simultaneously recorded spike-LFP signals was computed using a multitaper method implemented in Chronux 2.12 (see footnote 2) and the in-house MATLAB software (MathWorks, Natick, MA, United States). The Chronux coherence cept function (Bokil et al., 2010) was used to calculate the coherence value using spikes and synchronous LFPs at bands of 0.7–12, 12–35, 35–70, and 70–100 Hz. Compared to the control rats, alterations in the mean power frequency distributions were observed in these four frequency bands according to previously computed LFPs in the rats with dopaminergic lesions (Wang et al., 2016; Li et al., 2021). First, at least 5-min epochs were selected for analysis, during which simultaneously recorded spike-LFP signals were segmented into non-overlapping windows of equal length (5 s). Next, the multitaper method (9 tapers) was applied separately to compute the coherence for the current window. Final coherence value was estimated by calculating the average coherence over all windows, which ranged from 0 to 1. High coherence between spike-LFP signals indicates perfect synchronization, which correlates with fluctuations in power that are associated with oscillation amplitude and time (Sharott et al., 2005; Porter et al., 2020).

#### Phase Locking

Functional relationships between M1 and PF were also estimated by calculating phase locking. Phase locking is a measure of the consistent phase or amount of phase locking between two brain regions and correlates with oscillation fluctuations over time without considering any amplitude relationships. The same spike-LFP pairs that were used in the coherence analysis were included in the phase-locking estimation analysis.

First, phase distribution was described in the angular distributions of PF spikes in relation to their ongoing M1LFP activity. A third-order Butterworth filter was used to perform bandpass filtering for M1LFPs, and then the instantaneous phase relationship between the peak time of PF and the frequency band of M1LFPs at 0.7–12, 12–35, 35–70, and 70–100 Hz was calculated with the Hilbert transform (Nakamura et al., 2014; Sharott et al., 2017). Using this approach, phase histograms (20 bins) of spike timing relative to peaks in the M1 LFP correspond to a phase of 0° and troughs to a phase of 180° for each cell. Only PF neurons that contained at least 40 spikes were used for phase-locking estimation (defined as having *p* < 0.05 in Rayleigh’s uniformity test) to ensure that the statistical results were valid, of which the mean phase angle was only computed for each of the neurons that were significantly phase-locked. Differences between the mean phase angles were assessed by the Warson–Williams *F* test. Second, the length of the mean phase angle (range of to1, the closer to 1, the more preferred the angles) of the phase distribution was also computed (Abdi et al., 2015). It was used to describe and quantify the level of phase-locking around the mean angle for individual neurons. The difference between the lengths of the mean phase angle was analyzed by the Mann–Whitney *U* test. Subsequently, mean phase angle and mean vector length were calculated and displayed in circular plots using the Circular Statistics toolbox for MATLAB (Berens, 2009; Sharott et al., 2012). The line radiating from the center was used to indicate the vector and length of the mean phase angle in the circular histogram.

### Molecular Characterization of Recorded and Juxtacellularly Labeled Single Neurons

Previous studies have characterized the electrophysiological properties of two different subpopulations of PF neurons by performing *in vivo* extracellular recordings in dopamine-intact and hemiparkinsonian rats (Wang et al., 2016; Zhang et al., 2019b). An *in vivo* juxtacellular labeling method was used on anesthetized dopamine-intact rats to establish the consistency between electrophysiological properties and the immunohistochemical morphology of a subpopulation of PF neurons. Single neurons were selectively labeled with neurobiotin using the juxtacellular method as described previously (Pinault, 1996; Sharott et al., 2012). Briefly, a filamented borosilicate glass pipette (1.5 mm outer diameter, 0.86 mm inner diameter, Sutter Instrument) was pulled with a horizontal micropipette puller (P-97; Sutter Instrument) to a tip opening of 1-2 μm and resistance of 15–25 MΩ. Pipettes were filled with neurobiotin (2% w/v; Vector Laboratories, Burlingame, CA, United States) in a 1-M NaCl solution, and a chlorided silver wire was inserted and used as the recording and labeling microelectrode. Each rat was anesthetized with urethane (1.2–1.4 g/kg, i.p.) and a fixed stereotaxic apparatus (as described above). Recordings were performed in the PF at the following stereotaxic coordinates: anterior: –4.2 ± 0.1 mm from the bregma; lateral: ± 1.2 ± 0.1 mm; ventral: 6 ± 0.5 mm from the skull surface. Two holes were drilled in the bilateral PF on the skull. Neural signals were amplified with a multichannel bioamplification system (SWF-2 + RM6280BD; Chengdu, China), bandpass filtered at.16–3 kHz, and sampled at 20 kHz. Following successful characterization of isolated individual neuron spikes (i.e., single-unit activities), extracellular recordings were performed for 5–10 min for subsequent offline analysis using a standard “spike-sorting” procedure in Offline Sorter (Plexon, Inc., Dallas, TX, United States) as described above for identification and classification of neuron sections. Then, juxtacellular labeling of neurons in one hemisphere was performed by injecting a dye with 5–10 nA positive depolarizing rectangular pulses (duration: 0.2 s, 50% duty cycle) at 2.5 Hz through the recording electrode. The optimal position of the electrode was identified when the firing pattern of a neuron was robustly modulated by the current injection. Neuronal firing was modulated by microelectrophoresis current for at least 2 min.

### Immunochemistry

Within 2–4 h after juxtacellular labeling, the rats were sacrificed with an overdose of urethane (3 g/kg, i.p.) and perfused *via* the ascending aorta with 100 ml of saline (0.9% NaCl) followed by 300 ml of a fixative solution (4% paraformaldehyde in PBS). The rats were decapitated, and the brains were post-fixed with a paraformaldehyde solution overnight and subsequently immersed in 30% sucrose in PBS for 48 h at 4°C until they descended to the bottom of glass bottles. Then, the brains were removed and cut into slices at a 50-μm thickness.

First, sections were stained for the location of neurobiotin-labeled neurons using Cy2-conjugated streptavidin [1:1,000 dilution; Jackson ImmunoResearch Laboratories; catalog# 136724 (year 2019)], and then relevant sections containing neurons were further incubated for dual immunofluorescence staining to identify glutamatergic (monoclonal anti-vesicular glutamate transporter 1 antibody, 1:1,000 dilution; Millipore; catalog# 5502), GABAergic [monoclonal anti-glutamate decarboxylase antibody, 1:1,000 dilution; Millipore; catalog# 5406 (year 2019)] or parvalbumin [monoclonal anti-parvalbumin antibody, 1:1,000 dilution; Millipore; catalog# 1572 (year 2018)] neurons, and all sections were labeled with the secondary anti-mouse IgG-TRITC antibody [1:500 dilution; Abcam; catalog# ab6817 (year 2019)]. Juxtacellularly labeled neurons were photographed at 400 × magnification using a laser scanning confocal microscope (TCS SPE; Leica, Germany), and single juxtacellularly labeled neurons were imaged at 400 × magnification with the LAS AF series 009 software (Leica, Germany) and analyzed with the Image-Pro Plus software (version 5.1; Media Cybernetics, Inc., United States).

### Viral Vector-Mediated Transduction and Anterograde or Retrograde Tract-Tracing

We used a recombinant adeno-associated virus (AAV2/9-GFP) vector [titer, 1.6 × 10^12^ viral genomes (vg/ml)] as an anterograde viral vector tracer and AAV2/retro-GFP (titer, 1.3 × 10^12^ vg/ml) as a retrograde viral vector tracer and injected them into the parafascicular nucleus (PF) to investigate the potential anatomical connectivity between the PF and M1 and observe anterograde and retrograde transneuronal spread of neural tracers within the same pathway. Both anterograde and retrograde neural tracers containing a gene-encoding enhanced green fluorescent protein (GFP) were obtained from Hanbio (Hanbio Biotechnology Co., Ltd, Shanghai) and used as described previously (Sun et al., 2021).

For localized *in vivo* virus delivery, adult male Wistar rats weighing 290–310 g were separated into two groups. The animals were anesthetized with urethane (1.2 mg/kg, Sigma, St. Louis, MO, United States) and then placed in a stereotaxic frame (as described above). The scalp was shaved, and a small incision was made along the midline to expose the skull. After leveling the head relative to the stereotaxic frame, injection coordinates based on the reference (Paxinos and Watson, 2007) were used to mark the location on the skull directly above the unilateral PF (–4.2 ± 0.1 mm posterior to the bregma, 1.2 ± 0.1 mm lateral to the sagittal suture, and 6 ± 0.5 mm from skull surface), and a small hole (0.5 mm diameter) was drilled. Viruses were delivered through a glass microinjection capillary (1–1.5 μm tip diameter, 10–15 MΩ; Sutter Instruments, Novato, CA, United States) pulled with a glass electrode puller (P-97, United States) to have a tip inner diameter of 15 mm using a Stoelting apparatus pressure injection pump (789311S, United States). For anterograde tracing of PF projections, we infused anterograde AAV2/9-GFP vectors (0.4 μl, 1.6 × 10^12^ vg/ml) to anatomically label projection neurons in the PF. The PF was also infused with AAV2/retro-GFP (0.4 μl, 1.3 × 10^12^ vg/ml) to retrogradely label projection neurons with fluorophores. The optimal doses and phases between the injection of each viral vector and perfusion of targeted sites were determined according to our previous research (Sun et al., 2021). Original titers were used for each virus injection. The total injection volume was 0.4 μl at a rate of 0.02 μl/min. Following injection, the micropipette remained in place for 10 min to minimize diffusion of virus into the pipette path. After the injection was complete, the incision was sutured, and the long-acting analgesic carprofen (5 mg/kg, Sigma, St. Louis, MO, United States) was injected subcutaneously to minimize inflammation and discomfort. The animals were allowed to fully recover from the anesthesia on a heating pad at 37°C.

After allowing 3 weeks (anterograde viral vectors) or 2 weeks (retrograde viral vectors) for neuronal transduction and labeling with GFP, the animals were euthanized and transcardially perfused with 100 ml of phosphate-buffered saline (PBS), followed by 300 ml of 4% w/v PFA in PBS. Brains were incubated overnight with fixative at 4°C and then subsequently immersed in 30% sucrose at 4°C before sectioning. Coronal sections were cut from each brain using a vibrating microtome, collected in series in PBS, and mounted on glass slides for examination under a microscope. Sequential coronal sections (50 μm thick) through the PF and M1 were cut from the brains of individual animals between the quantified sections for PF (–3.84 to –4.44 mm from the bregma) and M1 (–1.72 to 1.08 mm from the bregma), respectively. Selected photomicrographs for displaying fluorescence labeling were captured with a laser scanning confocal microscope (Leica TCS SPE; Leica, Germany) and MIRAX SCAN (3DHISTECH, Budapest, Hungary). For each chosen photomicrograph, a region of interest (ROI) was manually drawn around the target sites to isolate the area in the image for semiautomated analysis. We used a semiautomated analysis procedure to calculate the average fluorescence intensity of infected neurons in imaged sagittal sections.

### Histology

**FIGURE 2:**
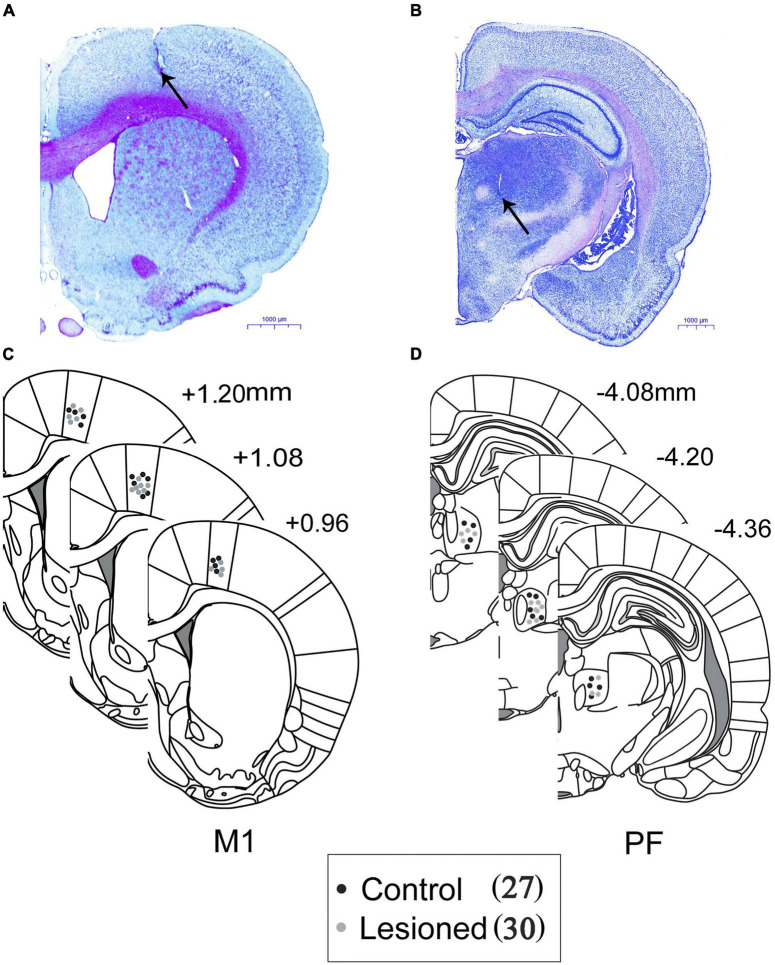
Histological representative example of Nissl-stained coronal sections of rat brain, photographed demonstrating the recording electrodes in the **(A)** M1 and **(B)** PF. Locations of electrode tips as marked by electrolytic lesions are marked with black arrows. Scale bar = 1,000 μm. Three schematic reconstructions in the **(C)** M1 and **(D)** PF of the three regions from the rat brain atlas of Paxinos and Watson represent the placements of electrodes in the DA-lesioned rats (gray circles) and control rats (black circles).

At the end of the last recording session, the rats were deeply anesthetized, and placement of each of the PF and M1 recording sites was marked by administering an electrolytic current (10 μA anodal current, 10 s × 3). The rats were intracardially perfused with 200 ml of saline followed by 200 ml of 4% paraformaldehyde and 1% potassium ferricyanide in PBS. Brains were post-fixed with a paraformaldehyde solution overnight and then immersed in 30% sucrose in PBS. Coronal sections of 40 μm were used for Nissl staining for structural identification and electrode tip verification *via* the deposited iron and/or electrolytic lesion (Figure 2). Only rats in which the tip of the electrode was confirmed to be correctly positioned in layer 5 of the M1 (Figure 2A) and PF (Figure 2B) were used for further analyses. Three schematics show the sites of recording electrodes in the M1 (Figure 2C) and PF (Figure 2D) of both the control (black dots, *n* = 27) and dopamine-lesioned rats (gray dots, *n* = 30). Then, in each of the dopaminergic-lesioned animals, immunohistochemical staining with tyrosine hydroxylase was performed to identify the 6-OHDA-induced lesion of dopaminergic neurons in the substantia nigra pars compacta (mean 94% loss, range 82–98%). A detailed description of the process and representative images are provided in our previous articles (Geng et al., 2016a; Wang et al., 2016).

### Statistical Analysis

The basic statistical analysis is presented as means ± SEM using the statistical software package Sigma Stat (SPSS 18, United States). The threshold for significance was *p* < 0.05. Before statistical comparison, the Kolmogorov–Smirnov test was conducted to judge whether non-circular datasets were normally distributed (*p* ≤ 0.05 to reject). For normally distributed data, differences between the control and lesioned groups were determined by two-tailed Student’s *t*-tests; for all the other data, Mann–Whitney rank sum tests were conducted. If the data did not display a normal distribution, Friedman RM ANOVA with Dunnett’s *post hoc* comparison was performed.

For multiple group comparisons, we performed a Kruskal–Wallis ANOVA on ranks, with Dunn’s test for further *post hoc* definition of comparisons.

For circular statistics, we determined whether spike phase distributions were non-uniform around the circle or had a common mean direction by performing Rayleigh’s test on population spiking. Differences in mean phase angles of two groups of neurons were analyzed by the Watson–Williams *F* test. Differences between mean resulting vector lengths of two groups were analyzed by the Mann–Whitney *U* test.

## Results

Only rats with a successful apomorphine-induced rotation test and those in which the tips of the electrode were confirmed to be positioned in layer 5 of the M1 and PF were used for further statistical analyses. Ten of the 40 rats were excluded: 1 rat did not learn to reach for food using its forepaw, two rats did not show adequate rotation behavior in the apomorphine-induced rotation test, the recording electrodes were misplaced in two rats, and five rats died during the experiment. Ultimately, 30 rats were used for analyses. Extracellular activities (spikes and LFPs) were analyzed synchronously from layer 5 of the M1 and PF *in vivo* when the 6-OHDA-treated rats (*n* = 16) and control rats (*n* = 14) were at rest and performed the voluntary forelimb-movement task.

### Effects of the 6-OHDA-Induced Lesion on Spiking and Local Field Potentials Activities in the Parafascicular Nucleus

**FIGURE 3:**
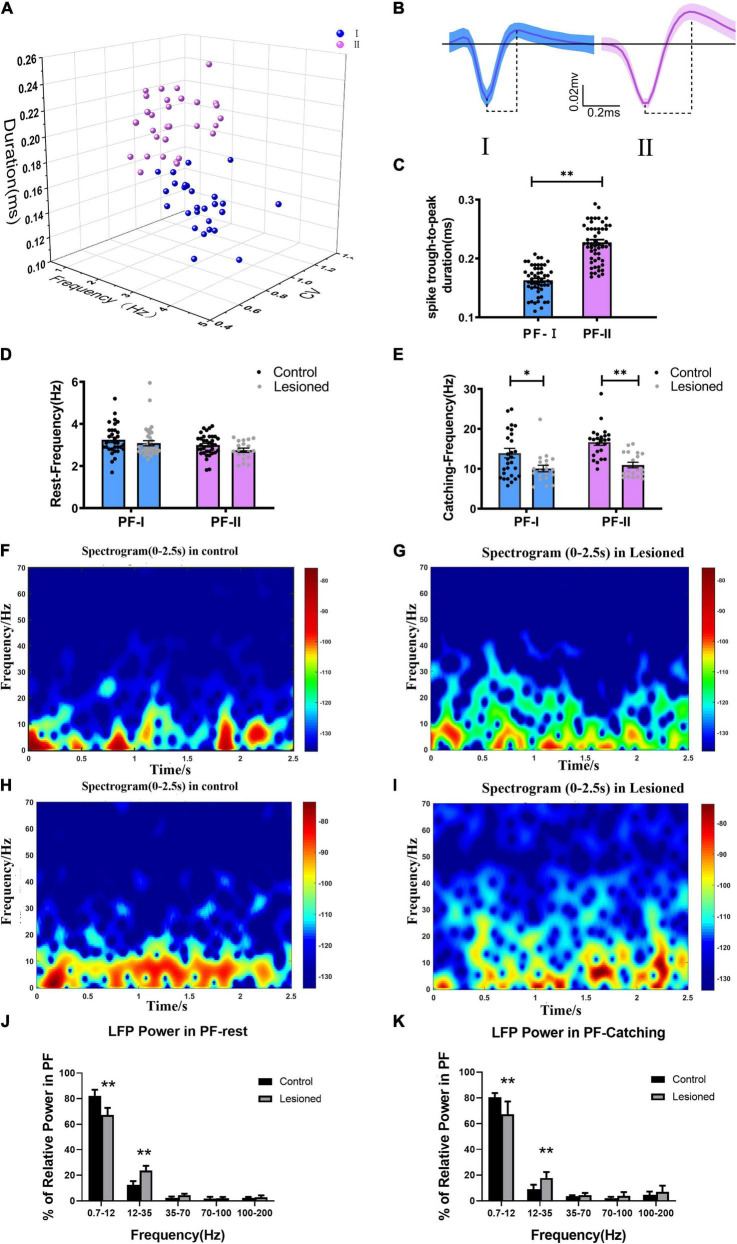
Neuron classification in the PF and alterations in firing rate and LFP power for the two types of neurons. **(A)** Corresponding cluster analysis of all recorded neurons according to duration and firing properties in 3D view. **(B)** Waveforms of the two types of neurons in the PF (blue, type I; purple, type II). The dotted lines below the waveforms show their trough-to-peak durations. **(C)** Statistical results of the through-to peak durations of all recorded neurons between the two subtypes of neurons were different. **(D)** The bar and scatter plot showed the mean frequency from type I (the blue bar) neurons and type II neurons (the purple bar) during resting and **(E)** catching movement states respectively, with black dots for the control rats and gray dots for the 6-OH DA-treated rats. Spectrograms of LFP power from the PF in the **(F)** control rats and **(G)** lesioned rats during resting states. Spectrograms of LFP power from the PF in the **(H)** control rats and **(I)** lesioned rats during catching movement states. **(J)** Bar graphs show the mean relative LFP power from different frequency bands between the control rats (black bars) and the 6-OH DA-treated rats (gray bars) during resting and **(K)** catching movement states, respectively. Error bars represent SEM, * indicates *p* < 0.05. ^**^ indicates *p* < 0.01.

Based on the widely accepted electrophysiological specificity of spike waveform characteristics (Minamimoto and Kimura, 2002; Beatty et al., 2009) and our previous study (Wang et al., 2016), the 67 recorded PF neurons (from 13 control rats) were classified into two subgroups based on discharge electrophysiological properties, including cluster analysis, spike waveform, and spike trough-to-peak duration of action potentials, i.e., PF-I and PF-II (Figures 3A–C). The PF-I subtype (*n* = 32, blue) had a low-amplitude, narrow trough-to-peak duration (0.16 ± 0.02 ms) and high-frequency tonic activity. The PF-II subtype (*n* = 35, purple) featured a high-amplitude, long trough-to-peak duration (0.23 ± 0.04 ms) and low-frequency activity. Next, we defined how chronic depletion of dopamine alters the electrical activity of the PF two subgroups at the level of single neurons (spike) and small neuronal ensembles (LFP) during rest and catching periods. During the resting state, the mean firing rate of PF-I neurons was not significantly different (3.09 + 0.12 vs. 3.25 + 0.13, *p* = 0.37) between the lesioned group (*n* = 36 neurons from 14 rats) and control group (*n* = 32 neurons from 13 rats); the difference in PF-II neurons between the lesioned group (2.75 + 0.10 vs. 3 + 0.09, *p* = 0.08) (*n* = 19 neurons from 14 rats) and the control group (*n* = 35 neurons from 13 rats) was not significant (Figure 3D). However, compared to the control group, both PF-I and PF-II neurons in the 6-OHDA-treated group showed a lower firing rate during catching movement periods (10.06 + 0.83 vs. 13.92 + 1.2, *p* = 0.01 and 10.94 + 0.70 vs. 16.67 + 0.81, *p* = 0.001, respectively) (Figure 3E).

In summary, chronic dopaminergic neuron lesions result in alterations in PF subtype neuronal spike activities specifically during the epoch of catching food movement rather than inattentive rest.

In addition, dopamine depletion correlated with alteration in special frequencies of LFPs during rest and catching movements. LFP activities in the PF in each 10-s period of continuous recording were divided into four frequency bands (0.7–12, 12–35, 35–70, 70–100 Hz, and 100–200 Hz) as described in our previous studies (Wang et al., 2016; Li et al., 2021).

Compared with the control group, a significant difference was observed in the PF-LFP power in the 6-OHDA-treated group. Based on a visual inspection, time-frequency spectrograms of the lesioned rats (Figures 3G,I) showed that the power in the low-frequency band was decreased, but that power was increased in the high-frequency band compared to the control rats (Figures 3F,H). During rest, the 0.7–12 Hz relative power was significantly reduced in the 6-OHDA-treated group (*n* = 14 rats) compared with the control group (*n* = 13 rats) (67.27 ± 2.27% vs. 82.26 ± 1.96%, *p* < 0.01). However, the 12–35 Hz relative power was increased in the 6-OHDA-treated group compared with the control group (23.71 ± 1.49% vs. 12.46 ± 1.27%, *p* < 0.01) (Figure 3J). During catching movements, the 0.7–12 Hz relative power was significantly decreased in the 6-OHDA-treated group (*n* = 14 rats) compared with the control group (*n* = 13 rats) (67.26 ± 4.1% vs. 80.49 ± 0.99%, *p* < 0.01). However, the 12–35 Hz relative power was significantly increased in the 6-OHDA-treated group compared with the control group (17.71 ± 1.91% vs. 9.02 ± 1.07%, *p* < 0.01) (Figure 3K). In summary, our results simultaneously indicated that PF-LFP powers during rest and catching movement periods were obviously disrupted by dopaminergic neuron lesions.

### Effects of 6-OHDA-Induced Lesions on Spiking and Local Field Potentials Activities in M1

**FIGURE 4:**
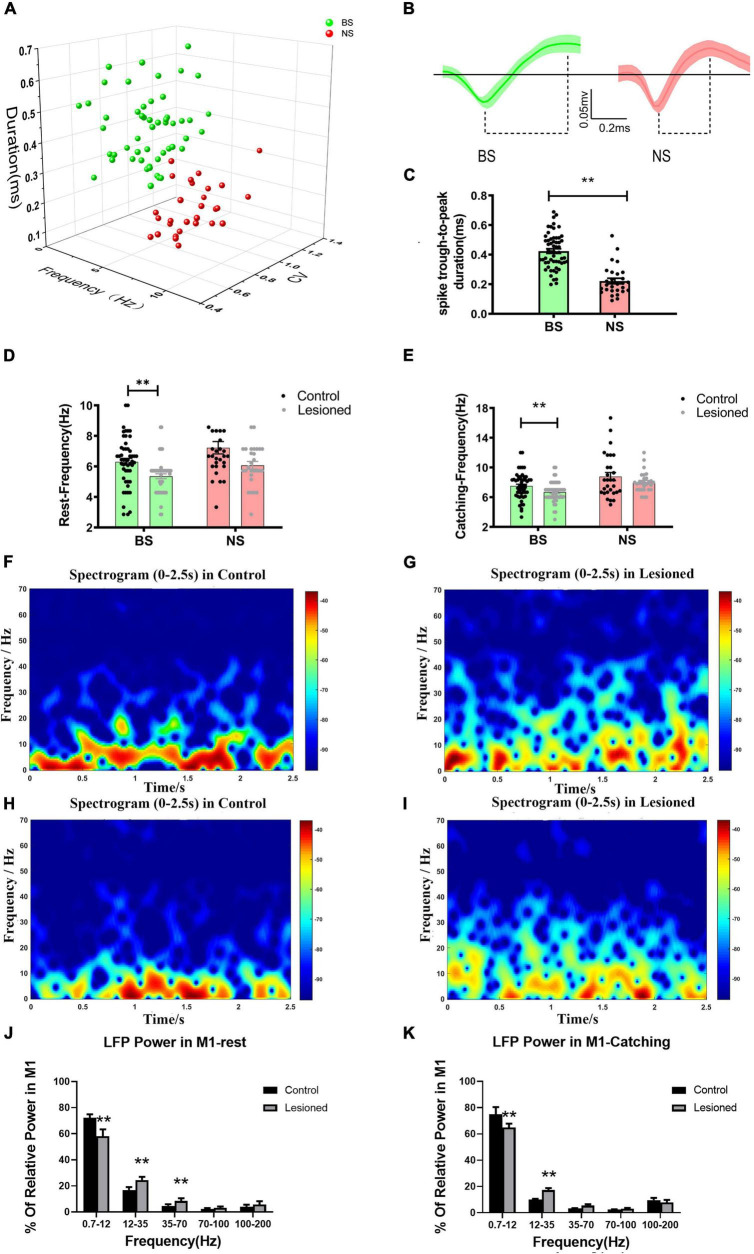
Neuron classification in the M1 and alterations in firing rate and LFP power for the two types of neurons. **(A)** Corresponding cluster analysis of all recorded neurons according to duration and firing properties in 3D view. **(B)** Waveforms of the two types of neurons in M1 (green, broad spike, BS; red, narrow spike, NS). Dotted lines below the waveforms show their through-to peak durations. **(C)** Statistical results of the through-to peak durations of all recorded neurons between the two subtypes of neurons were different. **(D)** The bar and scatter plot showed the mean frequency from BS (the green bar) neurons and NS neurons (the red bar) during resting and **(E)** catching movement states respectively, with black dots for the control rats and gray dots for the 6-OH DA-treated rats. Spectrograms of LFP power from the M1 in the **(F)** control rats and the **(G)** lesioned rats during resting states. Spectrograms of LFP power from the M1 in the **(H)** control rats and the **(I)** lesioned rats during catching movement states. **(J)** Bar graphs showed the mean relative LFP power from different frequency bands between the control rats (the black bars) and the 6-OHDA-treated rats (the gray bars) during resting and **(K)** catching movement states respectively. Error bars represent SEM, and ** indicates *p* < 0.01.

As described for the distinguished different neuronal subtypes mentioned above in the PF, we used the same methods to detect and sort spiking neurons in the M1 based on widely accepted spike waveform characteristics (Isomura et al., 2009; Kaufman et al., 2013; Saiki et al., 2014). Each sorted single unit was discriminated based on waveform shape, 2-/3-dimensional (2D/3D) principal component (PC) clusters, ISIs, and spike duration width (trough-to-peak measure), and defined in the same manner as described in our previous studies (Geng et al., 2019; Li et al., 2021). We sorted the 81 recorded M1 neurons (from 14 control rats) into two subgroups: putative pyramidal neurons and putative interneurons. The putative pyramidal neurons exhibited broad spike (BS, green, *n* = 52) widths and asymmetric waveforms, with higher spike amplitudes and both regular and burst-like firing patterns. The putative interneurons exhibited narrow spike (NS, red, *n* = 29) widths with lower spike amplitudes and a random firing pattern. The comparison of two sorted neuronal types present in the two subgroups was identified in 3D PC clusters (Figure 4A) and spike waveforms (Figure4B), and spike duration (trough-to-peak measure) was significantly longer for BS neurons (0.45 ± 0.03 ms) than for NS neurons (0.24 ± 0.02 ms, *p* < 0.001) (Figure 4C).

Next, we defined how chronic depletion of dopamine altered the electrical activity of two M1 subgroups at the level of single neurons (spike) and small neuronal ensembles (LFP) during rest and catching periods. In the resting state, the mean frequency of BS neurons in the lesioned rats (*n* = 52 neurons from 16 rats) was significantly reduced (5.36 ± 0.16 vs. 6.31 ± 0.22, *p* = 0.001) compared with the control rats (*n* = 52 neurons from 14 rats); however, the frequency of NS neurons in the lesioned rats (*n* = 28 neurons from 16 rats) showed no changes (6.07 ± 0.25 vs. 7.22 ± 0.41, *p* = 0.1) compared with the control rats (*n* = 26 neurons from 14 rats) (Figure 4D). In catching periods, the firing rate of BS neurons in the lesioned rats (*n* = 52 neurons from 16 rats) was also significantly lower (6.68 ± 0.18 vs. 7.51 ± 0.25, *p* = 0.009) than that in the control rats (*n* = 52 neurons from 14 rats); however, the frequency of NS neurons in the lesioned rats (*n* = 28 neurons from 16 rats) showed no change(8.12 ± 0.26 vs. 8.84 ± 0.52, *p* = 0.22) compared with that in the control rats (*n* = 26 neurons from 14 rats) (Figure 4E).

In summary, chronic dopaminergic neuron lesions induced significantly different effects on the firing properties of presumed pyramidal neurons (BS) and interneurons (NS). The BS neurons displayed reduced firing rates, while the NS neurons were not affected in either resting or catching conditions.

At the same time, dopamine depletion correlated with alteration in special frequencies of M1-LFP activities. As shown in the time-frequency spectrogram, the power spectrograms of LFPs from the M1 of the lesioned (Figures 4G,I) and control rats (Figures 4F,H) exhibited relatively distinct patterns of frequencies. During rest, LFP activities were analyzed using samples of 10-s segments from the raw data. Most notably, significantly lower relative power from the dopamine-lesioned group (*n* = 16 rats) was detected in the 0.7–12 Hz range compared to the control group (*n* = 14 rats) (58.1 ± 2.14% vs. 72.3 ± 1.04%, *p* < 0.01), while the power in 12–35 Hz and 35–70 Hz ranges was significantly larger in the dopaminergic lesion group than in the control group (24.55 ± 0.97% vs. 16.88 ± 0.91%, *p* < 0.01 and 8.46 ± 0.84% vs. 4.69 ± 0.51%, *p* < 0.05, respectively) (Figure 4J). During catching movements, the dopaminergic lesion group (*n* = 16 rats) showed a lower relative power in the 0.7–12 Hz band than the control group (*n* = 14 rats) (64.89 ± 2.96% vs. 74.95 ± 5.49%, *p* < 0.01), while the relative power at 12–35 Hz was significantly increased in the dopaminergic lesion group compared with the control group (17.31 ± 1.47% vs. 10.01 ± 0.65%, *p* < 0.01) (Figure 4K). In summary, our results confirmed that M1-LFP powers during rest and catching movement periods were obviously disrupted by chronic dopaminergic neuron lesions.

### Neurochemical and Electrophysiological Properties of Neurons in the Parafascicular Nucleus

**FIGURE 5:**
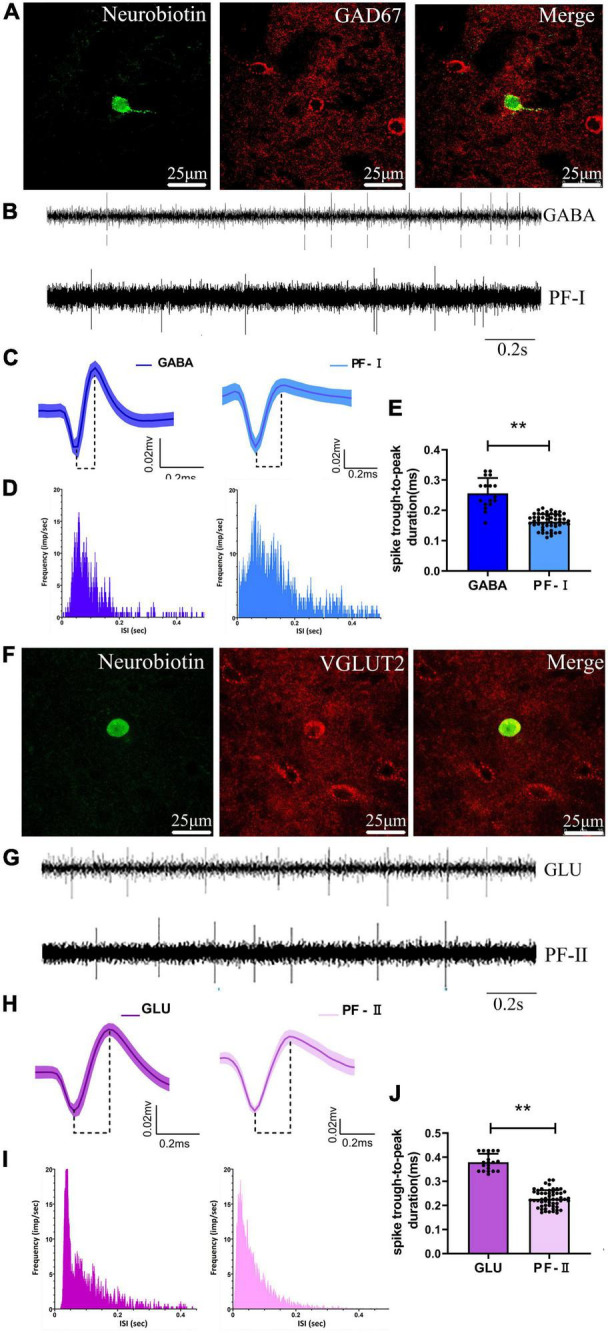
Identified neurochemical and electrophysiological properties of PF neurons and comparison with microwire electrode characteristics. A representative fluorescent image of immuno-labeled GABA-positive neuron in the **(A)** PF, and of immuno-labeled GLU-positive neuron in the PF **(F)**. Compare examples of **(B)** baseline firing properties, **(C)** average extracellular waveform, and **(D)** interspike interval (ISI) distribution for the labeled γ-aminobutyric acid (GABA)ergic-positive neuron and microwire electrode recording PF-Ineurons, respectively. Comparison of examples of **(G)** baseline firing properties, **(H)** average extracellular waveform, and **(I)** ISI distribution for the labeled glutamatergic (GLU)-positive neuron and s electrode recording PF-II neurons, respectively. A significant difference in spike duration (trough-to-peak measure) both for the **(E)** GABA-positive neuron with PF-Iand the **(J)** GLU-positive neuron with PF-II neurons, respectively. **p < 0.001.

We recorded and juxtacellularly labeled 37 neurons in the PF of anesthetized rats *in vivo* using a glass microelectrode, and were subsequently identified by their neurobiotin-labeled immunofluorescence signals and confirmed to be γ-aminobutyric acid (GABA)-ergic-positive (*n* = 17) or glutamatergic (GLU)-expressing (*n* = 20) neurons (Figures 5A,F). An additional double GABAergic labeling experiment was performed to further investigate the presence of GABAergic neurons in the PF (Supplementary Figures 1A,B). Based on the recording of baseline firing properties, waveform characteristics, firing frequency, and ISI distribution, two electrophysiologically distinct subgroups of PF neurons were identified with an off-line sorter that are similar to the subgroups of PF neurons electrophysiologically recorded with microwire electrodes (see details in section “Identification and classification of neurons,” identification and classification of neurons). GABA-positive neurons were electrophysiologically characterized as PF-I neurons identified with microwire electrodes recording the baseline signal recording firing rate and pattern, waveform, and ISI distribution, i.e., the “match signals” (Figures 5B–D), and GLU-positive neurons were characterized as PF-II neurons identified with microwire electrode recordings, i.e., the “match signals” (Figures 5G–I). However, significant differences in spike duration (trough-to-peak measure) were observed for the GABA-positive neurons (0.26 ± 0.01, *n* = 17 neurons) when the data were compared with the same spikes recorded using microwire electrodes (0.16 ± 0.02, *n* = 32 neurons, *p* < 0.01) (Figure 5E) and for the GLU-positive neurons (0.38 ± 0.01, *n* = 20 neurons) when compared with the same spikes recorded using microwire electrodes (0.23 ± 0.04, *n* = 35 neurons, *p* < 0.01) (Figure 5J). The potential explanation is that standard bandpass filtering potentially alters the shape of these extracellularly recorded action potentials, as described in a previous study (Brown et al., 2009). We speculate that the discrepancy might also differentially contribute to the diameter of the recording electrode tips, but future experiments will be required to test this hypothesis. In summary, these data indicate that either GABA neurons are PF-I neurons or GLU neurons are PF-II neurons based on their neurochemical and electrophysiological properties.

### Identification of Neurochemical and Electrophysiological Properties of Neurons in M1

**FIGURE 6:**
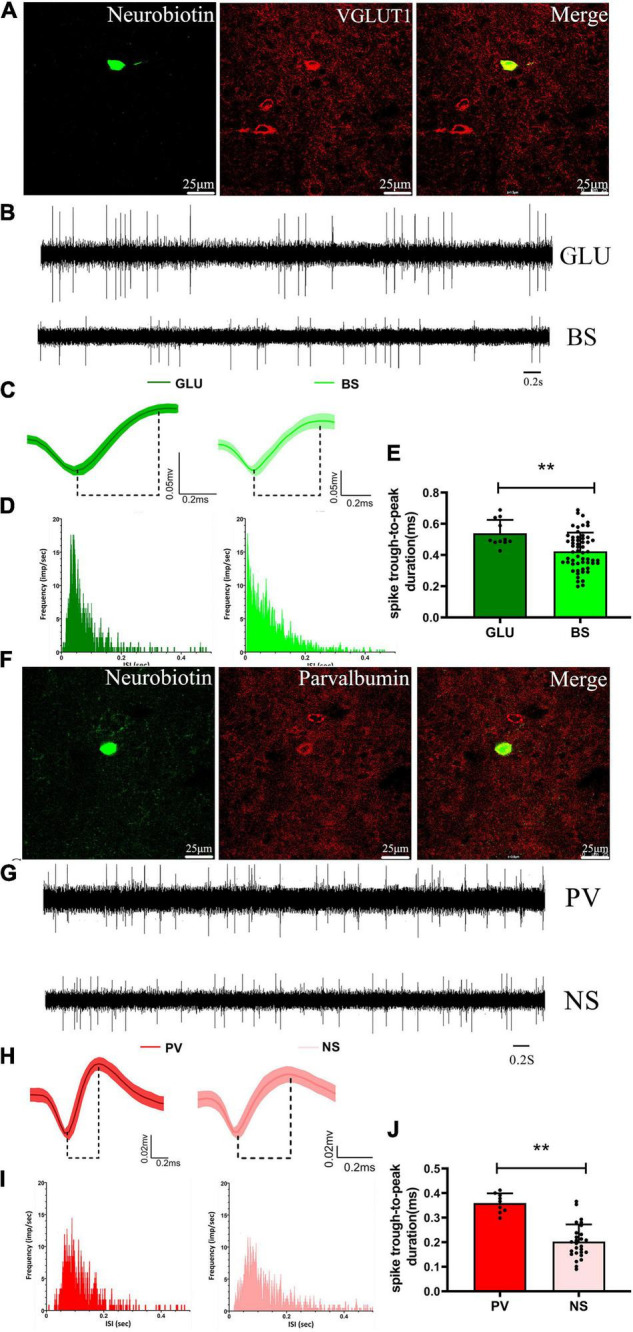
Identified neurochemical and electrophysiological properties of M1 neurons and comparison with microwire electrode characteristics. A representative fluorescent image of immuno-labeled GLU-positive neuron in the M1 **(A)**, and of immuno-labeled parvalbumin-positive neuron in the M1 **(F)**. Compare example of **(B)** baseline firing properties of **(C)** average extracellular waveform and **(D)** ISI distribution for the labeled GLU-positive neuron and microwire electrode recording BS neurons, respectively. Compare example of **(G)** baseline firing properties of **(H)** average extracellular waveform and **(I)** ISI distribution for the labeled parvalbumin-positive neuron and microwire electrode recording NS neurons, respectively. A significant difference in spike duration (trough-to-peak measure) both for the **(E)** GLU-positive neuron with BS and the **(J)** parvalbumin-positive neuron with NS neurons, respectively. **p < 0.001.

We recorded and juxtacellularly labeled 20 neurons in the M1 of anesthetized rats *in vivo* using a glass microelectrode, which were subsequently identified by their neurobiotin-labeled immunofluorescence signals and confirmed to be GLU-positive (*n* = 11) or parvalbumin (PV) (*n* = 9) neurons (Figures 6A,F). Based on the recording of baseline firing properties, waveform characteristics, firing frequency, and ISI distribution, two electrophysiologically distinct subgroups of M1 neurons were identified with an off-line sorter, which are similar to the subgroups of M1 neurons electrophysiologically recorded with microwire electrodes (see details in section “Effects of 6-OHDA-induced Lesions on Spiking and LFP activities in M1”). The GLU-positive neurons were electrophysiologically characterized as BS neurons identified with microwire electrode recordings of baseline signal recording firing rate and pattern, waveform, and ISI distribution, i.e., the “match signals” (Figures 6B–D), and the PV-positive neurons were characterized as NS neurons identified with microwire electrode recordings, i.e., the “match signals” (Figures 6G–I). However, significant differences in spike duration (trough-to-peak measure) for the GLU-positive neurons (0.54 ± 0.03, *n* = 11 neurons) were observed compared with the same spikes recorded using microwire electrodes (0.42 ± 0.02, *n* = 52 neurons, *p* < 0.01) (Figure 6E) and for the PV-positive neurons (0.36 ± 0.01, *n* = 9 neurons) compared with the same spikes recorded using microwire electrodes (0.22 ± 0.02, *n* = 27 neurons, *p* < 0.01) (Figure 6J). In summary, these data indicate that either the GLU neurons are BS neurons or the PV neurons are NS neurons based on their neurochemical and electrophysiological properties.

### Effect of DA Loss on the Relationships Between Parafascicular Nucleus Spikes and M1 Local Field Potential

The results described above indicate significant alterations in neural activity in spike and LFP activities recorded in the PF and M1 following chronic dopamine depletion. Furthermore, studies have reported that dysregulation of interareal synchronization is widely distributed across the network and cannot be located in any individual structure. We next investigated how the activity of individual subgroups of PF neurons varied over time with respect to ongoing M1 LFPs at different frequencies during rest or movement and whether these different neuronal subgroups have specific roles in shaping distinct LFPs in rats with chronic dopamine depletion.

The mean spike-LFP coherence value and phase lock were analyzed to evaluate the alteration relationship between the two subtypes of PF spikes and M1-LFPs (0.7–12, 12–35, 35–70, and 70–100 Hz) after DA loss during rest and catching movement epochs. Microwire electrodes were used for recordings in the DA-lesioned rats (*n* = 14) and control rats (*n* = 13).

**FIGURE 7:**
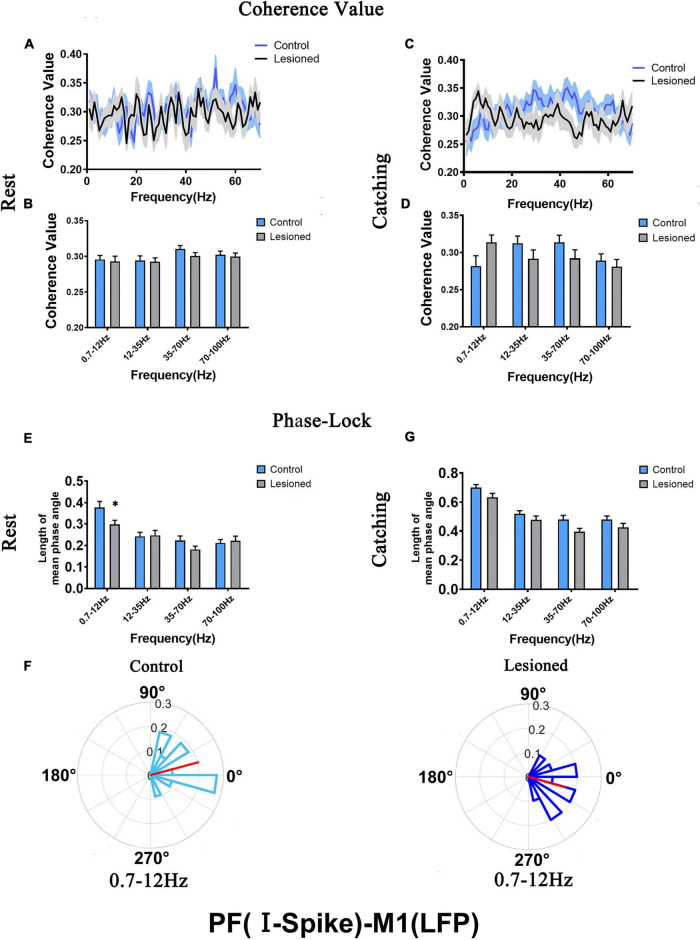
Coherence values and phase-lock of PF-I with respect to M1-LFPs in the control rats and the DA lesioned rats. Coherence values between PF-I (*n* = 21 neurons from 14 lesioned rats, and *n* = 28 neurons from 13 control rats) and M1-LFPs have no alterations during **(A,B)** rest and **(C,D)** catching movement states. During the resting epoch, phase-locking levels represented by the length of mean phase angle were significantly lower only in the range of 0.7–12 Hz in the lesioned rats compared to the control rats. Red lines radiating from the center indicated the measure of the strength of concentration of the distribution of the mean phase angle of all recording PF-I. Thus, PF-I neurons in the lesioned rats (dark blue) were less phase-locked to M1-LFP activities at 0.7–12 Hz than those neurons in the control rats (light blue) **(E,F)**. During catching movements, PF-I neurons in the lesioned rats did not significantly change in mean vector length compared to that of neurons in the control rats **(G)**. Data are expressed as the mean ± SEMs (*p < 0.05).

For PF-I, we analyzed the mean spike-LFP coherence value and phase lock of 21 and 28 neurons in the lesioned and control rats, respectively, during rest and 19 and 27 neurons in the lesioned and control rats, respectively, during catching movements (Figure 7).

**FIGURE 8:**
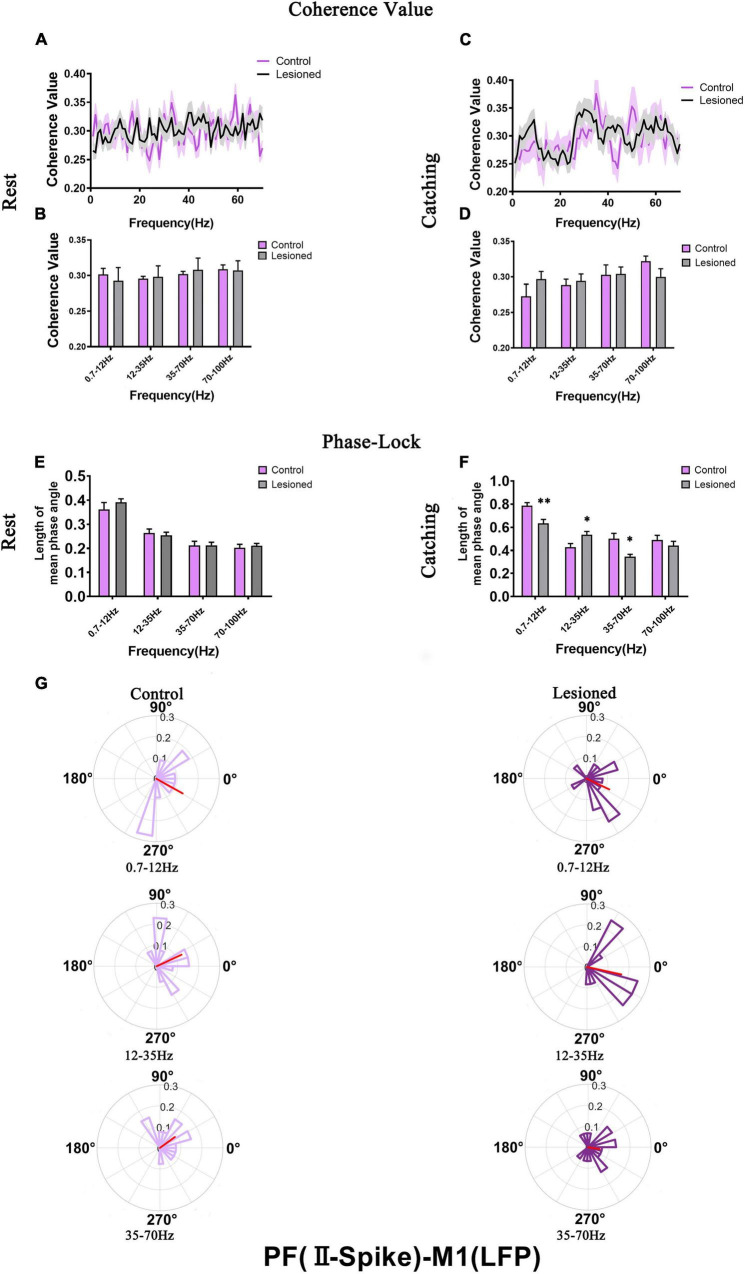
Coherence values and phase-lock of PF-II with respect to M1-LFPs in the control rats and the DA lesioned rats. Coherence values between PF-II (*n* = 19 neurons from 14 lesioned rats, and *n* = 30 neurons from 13 control rats) and M1-LFP have no alterations during **(A,B)** rest and **(C,D)** catching movement states. During the resting epoch, PF-II neurons in the lesioned rats did not significantly change in the length of mean phase angle compared to that of neurons in the control rats **(E)**. During catching movements, the length of mean phase angle was significantly lower in the ranges of 0.7–12 and 35–70 Hz in the lesioned rats (dark purple) compared to the control rats (light purple, but was significantly increased at 12–35 Hz **(F,G)**. Data are expressed as the mean ± SEMs (*p < 0.05, ^**^p < 0.01).

For PF-II, we analyzed the mean spike-LFP coherence value and phase lock of 19 and 30 neurons in the lesioned and control rats, respectively, during rest and 19 and 23 neurons in the lesioned and control rats, respectively, during catching movements (Figure 8).

For PF-I, the mean spike-LFP coherence value was not altered during either rest or movement (Figures 7A–D). The phase lock (length of mean phase angle) only decreased in the 6-OH DA-treated group (dark blue) at 0.7–12 Hz during rest compared with the control group (light blue) (0.3 + 0.02 vs. 0.38 + 0.03, *p* = 0.04) (Figures 7E,F). The length of the mean phase angle showed no alterations during movements (Figure 7G). Taken together, the effect of DA on the activity of individual PF-I neurons with respect to ongoing M1 LFPs may be negligible.

For PF-II, no difference in mean spike-LFP coherence value was observed during either rest or movements (Figures 8A–D). The length of the mean phase angle was not altered during rest (Figure 8E). However, dopaminergic neuron depletion exerted remarkable effect on the length of the mean phase angle in PF-II neurons during catching movements. The length of the mean phase angle was conspicuously decreased in the 6-OH DA-treated group (dark purple) compared with the control group (light purple) at 0.7–12 Hz (0.63 + 0.03 vs. 0.79 + 0.03, *p* = 0.003) and 35–70 Hz (0.35 + 0.02 vs. 0.5 + 0.04, *p* = 0.018) but was significantly increased at 12–35 Hz (0.54 + 0.03 vs. 0.43 + 0.03, *p* = 0.03) (Figures 8F,G). Thus, PF-II neurons in the lesioned rats were more phase-locked to M1-LFP activities at 12–35 Hz than neurons in the control rats. In summary, dopaminergic neuron depletion exerted a remarkable effect on the activity of individual PF-II neurons with respect to ongoing M1 LFPs.

### Effects of DA Loss on the Relationships Between M1 Spikes and Parafascicular Nucleus Local Field Potential

**FIGURE 9:**
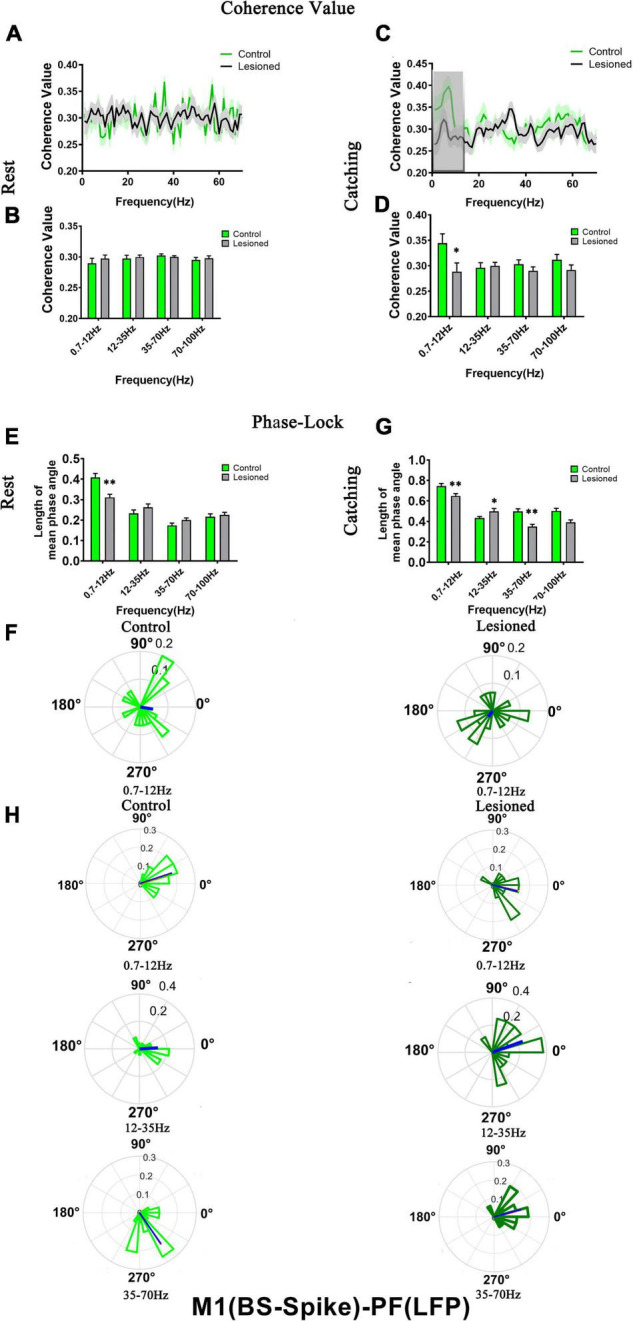
Coherence values and phase-lock of BS with respect to PF-LFPs in the control rats and the DA-lesioned rats. Coherence values between BS (*n* = 29 neurons from 16 lesioned rats, and *n* = 31 neurons from 14 control rats) and M1-LFP have no alterations during **(A,B)** rest states. During catching movements, coherence values have decreased in the lesioned group at 0.7–12 Hz compared with the control group **(C,D)**. During the resting epoch, phase-locking levels represented by the length of mean phase angle were significantly lower only in the range of 0.7–12 Hz in the lesioned rats compared to the control rats. Blue lines radiating from the center indicated the measure of the strength of concentration of the distribution of the mean phase angle of all recording BSs. The lower vector lengths indicated that BS neurons in the lesioned rats (dark green) were less phase-locked to PF-LFP activities at 0.7–12 Hz than those neurons in the control rats (light green) **(E,F)**. During catching movements, the length of mean phase angle was significantly lower in the ranges of 0.7–12 and 35–70 Hz in the lesioned rats (dark green) compared to the control rats (light green) but was significantly increased at 12–35 Hz **(G,H)**. Data are expressed as the mean ± SEMs (*p < 0.05, ^**^p < 0.01).

**FIGURE 10:**
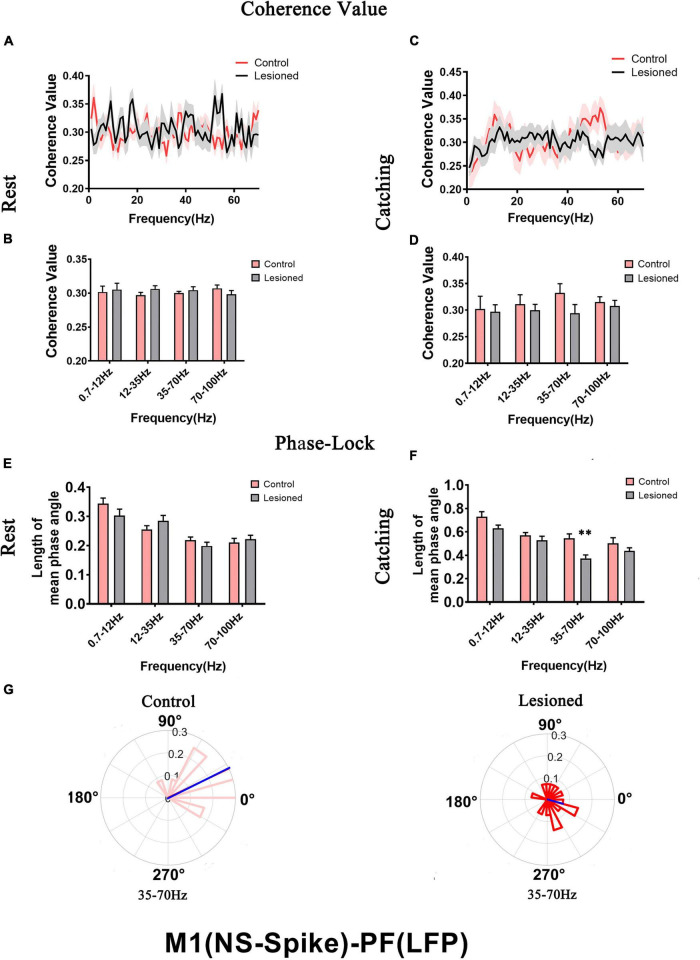
Coherence values and phase-lock of NS with respect to PF-LFPs in the control rats and the DA-lesioned rats. Coherence values between NS (*n* = 25 neurons from 16 lesioned rats, and *n* = 27 neurons from 14 control rats) and PF-LFP have no alterations during **(A,B)** rest and **(C,D)** catching movement states. During resting, NS neurons in the lesioned rats did not significantly change in the length of mean phase angle compared to that of neurons in the control rats **(E)**. Blue lines radiating from the center indicated the measure of the strength of concentration of the distribution of the mean phase angle of all recording NSs. During catching movements, the length of mean phase angle was significantly lower in the range of 35–70 Hz in the lesioned rats (dark red) compared to the control rats (light red) **(F,G)**. Data are expressed as the mean ± SEMs (^**^p < 0.01).

We next investigated how the activity of individual subgroups of M1 neurons varied over time with respect to ongoing PF LFPs at different frequencies during rest or movements and whether these different neuronal subgroups have specific roles in shaping distinct LFPs in rats with chronic dopamine depletion using the same analysis methods. Using microwire electrodes on the DA-lesioned rats (*n* = 16) and control rats (*n* = 14) to record BS neurons, we analyzed the mean spike-LFP coherence value and phase lock of 29 and 31 neurons in the lesioned and control rats, respectively, during rest and 27 and 29 neurons in the lesioned and control rats, respectively, during catching movements (Figure 9). For NS neurons, we analyzed the mean spike-LFP coherence value and phase lock of 25 and 27 neurons in the lesioned and control rats, respectively, during rest and 25 and 26 neurons in the lesioned and control rats, respectively, during catching movements (Figure 10).

For BS neurons, mean spike-LFP coherence value was not altered during rest (Figures 9A,B). The mean spike-LFP coherence value only decreased in the 6-OH DA-treated group at 0.7–12 Hz during catching movements compared with the control group (0.29 + 0.02 vs. 0.34 + 0.02, *p* = 0.04) (Figures 9C,D). For the phase-lock analysis, alterations were observed both in rest and catching movement epochs. During rest, the length of the mean phase angle of BS neuron spiking was significantly decreased in the 6-OHDA-treated group (dark green) compared with the control group (light green) in the 0.7–12 Hz range (0.31 ± 0.06 vs. 0.41 ± 0.02, *p* = 0.001) (Figures 9E,F). During the catching movement epoch, dopaminergic neuron depletion exerted a remarkable effect on the mean vector length in BS neurons. The mean vector length in BS neurons was conspicuously decreased in the 6-OHDA-treated group (dark green) compared with the control group (light green) at 0.7–12 Hz (0.65 + 0.02 vs. 0.74 + 0.03, *p* = 0.007) and 35–70 Hz (0.35 + 0.02 vs. 0.5 + 0.03, *p* = 0.002) but was significantly increased at 12–35 Hz (0.5 + 0.03 vs. 0.43 + 0.02, *p* = 0.04) (Figures 9G,H). Thus, dopaminergic neuron depletion had a remarkable effect on the activity of individual BS neurons with respect to ongoing PF LFPs.

For NS neurons, no difference in mean spike-LFP coherence value was detected during either rest or movements (Figures 10A–D). The length of mean phase angle was not altered during rest (Figure 10E). During catching movements, the length of the mean phase angle was conspicuously decreased in the 6-OHDA-treated group (dark red) compared with the control group (light red) at 35–70 Hz (0.37 + 0.03 vs. 0.55 + 0.04, *p* = 0.002 t) (Figures 10F,G). Thus, compared to BS neurons, dopaminergic neuron depletion had a negligible effect on the activity of individual NS neurons with respect to ongoing PF LFPs.

### Viral Vector Tracing of the Connectivity Between Parafascicular Nucleus and M1

**FIGURE 11:**
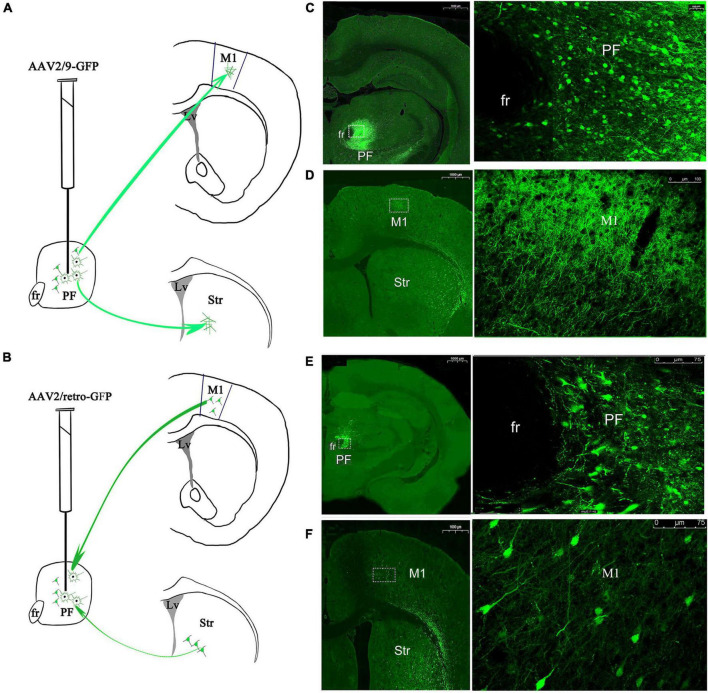
Immunofluorescent adenoviral vector tracers were stereotaxically injected into the rat PF to visualize afferent and efferent projections. Schematic of the procedures used to inject the **(A)** anterograde tracer AAV2/9-GFP and **(B)** retrograde viral vector tracer AAV2/retro-GFP. Expression of the anterograde tracer in PF neurons. The left lateral images are magnified views of the rectangles in the medial images, with cell bodies and dendritic neuropil of GFP-immunopositive neurons shown **(C)** and projecting to M1. The left lateral images are magnified views of the rectangles in the medial images, with only dendritic neuropil of GFP-immunopositive neurons shown **(D)**. Expression of the retrograde tracer in PF neurons. The left lateral images are magnified views of the rectangles in the medial images, with cell bodies and dendritic neuropil of GFP-immunopositive neurons shown **(E)**. Anterograde transport from the PF projects to axon terminal fields in the M1 and striatum. The left lateral images are magnified views of the rectangles in the medial images, with cell bodies and dendritic neuropil of GFP-immunopositive neurons detected in the M1 **(F)**. PF, parafascicular nucleus of the thalamus; fr, fasciculus retroflexus; M1, primary motor cortex; Str, striatum.

The data from our electrophysiological recordings provided evidence that dysregulated synchronization is diversely and widely distributed between the PF and M1 in rats with DA loss. Next, we injected immunofluorescence adenoviral vector tracers into the PF of rats to visualize afferent and efferent projections and to understand the potential anatomical connectivity between the PF and the M1. The optimal injection doses and time course of transfected tracers into the M1 were determined according to the dose–response curve generated in our recent study (Sun et al., 2021), i.e., 0.4 μl in 3 weeks for anterograde AAV2/9-GFP and 0.4 μl in 2 weeks for retrograde AAV2/9-GFP. Both the anterograde and retrograde viral vectors had the ability to infect PFneurons or project to the M1 (Figures 11A,B).

For animals infected with AAV2/9-GFP for 3 weeks, we observed a large area covering the transduction of GFP-positive fluorescent protein in the PF (relative intensity 804.21 ± 126.88 OD/μm^2^, mean ± SEM, *n* = 3 rats, 5–6 sections/rat), and the cell bodies and dendritic neuropil of GFP-immunopositive neurons were observed (Figure 11C). GFP-immunopositive axonal fibers from PF projection neurons were also observed in the M1; however, the density of axons with GFP + immunoreactivity was very weak and sparse, and no GFP-positive cell bodies were observed, revealing that PF axons were distributed in the M1 (Figure 11D).

For animals infected with the retrograde tracer AAV2/retro-GFP for 2 weeks, the cell bodies and dendritic neuropil that exhibited GFP immunoreactivity were densely observed in the PF (relative intensity 268.07 ± 84.59 OD/μm^2^, mean ± SEM, *n* = 3 rats, 5–6 sections/rat) (Figure 11E). In addition to the GFP-immunopositive axonal fibers from the PF, retrogradely labeled GFP immunoreactive somata were also observed in a small number of cortical neurons in the M1, revealing that the PF retrograde transported the viral vector into the projection area, i.e., the PF (Figure 11F). Generally, the AAV2/9- GFP-, AAV2/retro- GFP-, or AAV2/retro-GFP-mediated anterograde or retrograde viral vector tracers traveled ipsilaterally, because no fibers were observed in any areas of the contralateral side of the brain. Taken together, these data reveal ipsilaterally parallel afferent input and efferent output connectivity between the PF and the M1.

## Discussion

In this study, we clarify the patterns of altered interaction activity occurring across PF and M1 circuits following dopamine depletion during rat movements. We emphasize the selective entrainment and synchronization of neurons in the PF and M1, respectively, during abnormal LFP oscillations in cortical–thalamic circuits in parkinsonism. Compelling evidence shows that cell type-selective PF neurons exhibited aberrant firing rates and preferential and excessive phase-locked firing to cortical LFP oscillations mainly at 12-35 Hz (beta frequencies), consistent with the observation of the activity of M1 cell type-selective neurons with ongoing PF LFPs oscillation. However, this result also showed a decrease in phase-locking at 0.7–12 Hz and 35–70 Hz in PF and M1 circuits. In addition, this complicated functional reciprocal interaction was based on anatomical evidence for reciprocal connections from the PF to the M1. Collectively, the most notable finding was that the spike-LFP relationships in the PF and M1 circuits displayed excessive alterations, and that different PF or M1 neuronal subtypes may be responsible for orchestrating the synchronization of these pathological rhythms in PF and M1 circuits.

### Effect of DA Loss on Parafascicular Nucleus Spikes and Their Relationship With M1 Local Field Potentials

We identified two types of predominant neurons in the PF whose pathological alterations in extracellular electrical activities were correlated with distinct aspects during rest and movements in the 6-OHDA-lesioned rats. Changes in the neuronal firing rate and pattern in the PF from the lesioned rats are consistent with previous reports (Parr-Brownlie et al., 2009; Wang et al., 2016; Zhang et al., 2019b). Furthermore, anatomical and physiological evidence has shown that the CM/PF is severely degenerated in patients with PD (more than 30%) (Henderson et al., 2000a,b) and rats with a unilateral 6-OHDA-induced lesion of the substantia nigra (Sedaghat et al., 2009), combined with the fact that CM/PF deep brain stimulation alleviates some symptoms of PD (Smith et al., 2009). Importantly, this study provided evidence of dopamine dependency of the functionally defined PF in the firing of a subgroup of neurons that are differentially entrained to ongoing M1 LFP oscillations in hemiparkinsonian rats. Interestingly, during catching food movements, PF-II neurons are particularly prone to being recruited to abnormal functional connectivity between the PF and the M1.

Our recordings of individual neurons identified with the juxtacellular method collectively suggest that PF-II-type neurons coincide with glutamatergic-like neurons, which is similar to *in vivo* extracellular recordings (Zhu et al., 2019) and *in vitro* intracellular recordings (Beatty et al., 2009). These PF-II-type neurons displayed lower firing rates and preferentially aberrant phase-locked firing to M1 LFPs at 12–35 Hz (beta frequencies), with decreases in phase-locking at 0.7–12 and 35–70 Hz after dopamine depletion. The results are consistent with experiments showing a substantial and coherent alteration in special frequency LFPs in the motor nucleus of the thalamus and M1 of behaving 6-OHDA-lesioned rats (Brazhnik et al., 2016; Reis et al., 2019).

Conversely, PF-I neurons coinciding with GABAergic-like neurons were hypoactive but not synchronized to a large extent during cortical activation. Our study identified a few GABAergic-like neurons in the PF that were recognized by juxtacellular labeling and confirmed them with a later additional double GABAergic labeling experiment (Supplementary Figure 1). The presence of GABAergic neurons in the PF may suggest that local inhibitory circuits are formed by intrinsic GABAergic cells. We reasoned that this situation may contribute to shaping the output activity of thalamocortical projection neurons (Hirsch et al., 2015). Studies on PF GABAergic interneurons in rats and mice were almost excluded. However, previous studies have shown that no GABAergic interneurons were found in rats and mice except for a moderate amount contained in some sensory nuclei (Bentivoglio et al., 1991; Arcelli et al., 1997). This feature is not exclusive to a specific rodent, since other rodents (e.g., guinea pigs, hamsters, squirrels, etc.) contain numerous interneurons throughout this region (Arcelli et al., 1997). Researchers do not clearly understand to what extent these differences affect the complexity and distribution of thalamic interneurons across specific species (Jager et al., 2016). Nevertheless, some recent studies have identified a wider distribution of GABAergic interneurons in mice than previously reported. For example, a study revealed an unappreciated complexity of GABAergic interneurons in mouse thalamic nuclei, indicating that interneurons are not restricted to thalamic sensory nuclei but are present across modalities and hierarchical levels, including the PF (Jager et al., 2021). Another study showed that GABAergic neurons in the posterior PF receive structural and functional inhibitory connections from substantia nigra pars reticulata neurons and inhibit local glutamatergic PF neurons (Chen et al., 2020). Consistent with these studies, a single PF neuron, under the guidance of its firing feature, was selectively labeled with both neurobiotin and GABAergic immunostaining in our study. Then, a chemical phenotyping double labeling experiment proved the presence of GABAergic neurons in the PF. A limitation of this study is the lack of a corresponding relationship between the chemical phenotype of double-labeled neurons and juxtacellular-labeled neurons. A more detailed future analysis is required to establish a role for thalamic inhibitory interneurons in the PF in basal ganglia-thalamo-cortical circuits.

We also recorded some glutamatergic-like neurons recognized by juxtacellular labeling and immunochemistry with the anti-vesicular glutamate transporter vGLUT2 in the PF (similar results were obtained for vGLUT1 in the M1). As excellent markers of glutamatergic neurons, VGLUTs selectively package glutamate into synaptic vesicles and mediate glutamate transport (Pietrancosta et al., 2020). vGluTs are a distinct family of glutamate transporters that have been detected in axon terminals, and low levels of transporters are present in cell bodies and dendrites of glutamatergic neurons in certain brain regions. The presence of a particularly prominent punctuate, vGluT (axon terminal varicosities), immunoreactivity increased the difficulty of visualizing VGLUT-labeled neuronal somas (Kuramoto et al., 2011). However, some studies have provided evidence that few neurons in certain brain regions express the VGLUT protein and are capable of using glutamate as a neurotransmitter, such as the septum (Hajszan et al., 2004; Colom et al., 2005), ventral cochlear nucleus (Zhou et al., 2007), and anterodorsal thalamic nucleus (Oda et al., 2014). Similar to these studies, immunolabeling for the vGLUT2 protein was detected around cell bodies in the PF in this study. We speculated that after electrophysiological characterization, neurons were juxtacellularly labeled with neurobiotin, and that their somata were unequivocally identified, which may provide a reliable cytoarchitectonic reference for identifying neurons and facilitate the visualization of VGLUT2 labeling of neuronal somata. Moreover, we hypothesized that microiontophoretic current stimulation would change VGLUT2 accumulation in glutamatergic neuronal somata. Unfortunately, similar to the identification in GABAergic-like neurons, a limitation to be addressed is the lack of a triple immunofluorescence labeling technique to establish the colocalization of specific antibodies against neurobiotin and VGluT2 (as for vGLUT1 in the M1) and additional nuclear label.

The results from this study suggest that the spiking activity of PF neurons, similar to activities of some other neurons across the basal ganglia, exhibited cell type-selective locking to specific phases of cortical oscillations in parkinsonism (Sharott et al., 2018; Cagnan et al., 2019; Tinkhauser et al., 2020). However, our results emphasize the potential importance of aberrant, selective entrainment of the firing of a population of PF-II-type neurons, i.e., glutamatergic-like neurons recognized by recorded extracellular spiking and juxtacellular labeling technology, which are tightly correlated with the expression of exaggerated beta activity in thalamocortical circuits after dopamine loss. This result is consistent with the evidence of a strong effect of glutamatergic pathways on thalamic modulation underphysiological and pathological conditions (Sherman, 2014; Masilamoni and Smith, 2019). On the other hand, our results provide empirical evidence for a circuit-level mechanism of PD, particularly glutamatergic elements, revealing complex relationships between the thalamus and the cortex. The modulatory role of PF spikes has also been supported by anatomical evidence that the PF, a higher-order thalamic nucleus, receives major input from cortical layer 5 and cortical layer 6 and projects to the cerebral cortex to participate in thalamocortical circuits *via* branched axons (Sherman, 2016; Mandelbaum et al., 2019; Suzuki et al., 2021). Collectively, our results suggest that during chronic dopamine depletion, the PF continues to modulate the processing of information as a hub in thalamocortical circuits and is not limited to relaying information in the basal ganglia and processing other subcortical signals but may also be actively involved in information processing by integrating signals from the cortex.

### Effect of DA Loss on M1 Spikes and Their Relationship With Parafascicular Nucleus Local Field Potentials

The present results showed that dysfunction of the parkinsonian M1 is observed as (1) alterations in movement-related firing, (2) changes in certain LFP frequency oscillations (especially in 12–35 Hz, the beta band), and (3) functional reorganization of local circuits. First, after dopamine depletion, the presumptive pyramidal neurons showed reduced firing rates and altered firing patterns, while the presumptive interneurons were not significantly affected by dopamine depletion. This condition agrees well with studies showing that presumptive pyramidal neurons decrease their firing rate and display abnormal discharge patterns in parkinsonian animals (Pasquereau and Turner, 2011; Hyland et al., 2019; Rios et al., 2019; Li et al., 2021). Unfortunately, given the diversity and complexity of M1 interneurons (Naka and Adesnik, 2016), our understanding of how interneuron activity contributes to cortical functions and behaviors disturbed in parkinsonism is limited (Li et al., 2021). Accumulating evidence has shown that the M1 undergoes functional changes in individuals with PD, and M1 neurostimulation will undoubtedly improve in the future, as it permits neuron-type specific control and incorporates feedback from electrophysiological biomarkers measured locally (Underwood and Parr-Brownlie, 2021). Second, excluding the alteration of single spike activities, this study revealed that the M1 in the parkinsonian state was associated with alterations in certain LFP frequencies, especially those in the beta range (12–35 Hz). These data are partially consistent with a previous study in which an animal PD model showed a pathologically exaggerated beta range throughout cortico-basal ganglia circuits after dopamine depletion (Wang et al., 2015; Brazhnik et al., 2016; Cagnan et al., 2019; Geng et al., 2019). Third, our study highlights that chronic dopamine depletion alters not only the relationships of identified individual neuron PF spikes with M1 LFP activities but also the rhythmic synchronization between the identified individual neuron M1 spikes and PF LFP activities. Notably, recordings of putative pyramidal neurons and interneurons revealed that during catching food movements, dopamine depletion is associated with aberrant phase-locked firing to PF LFPs, with broad frequency bandwidths at 0.7–12, 12–35, 35–70, and 70–100 Hz. This finding was supported by a previous report that M1 pyramidal neurons are more vulnerable than other neurons to dopaminergic lesions (Pasquereau et al., 2015; Hyland et al., 2019; Rios et al., 2019; Crompe et al., 2020), and that pyramidal neuron sensitivity might be explained by direct input from thalamic nuclei (Sherman, 2017; Nashef et al., 2021), which themselves receive direct inputs from the BG and are affected by the propagation of abnormal BG activity in individuals with PD (Singh, 2018; Reis et al., 2019). However, we also found that putative interneurons (expressing parvalbumin) exhibited a decrease in phase-locking at 35–70 Hz, although the interneurons did not exhibit specific changes in the firing rate during catching food movement (Li et al., 2021), implying a possible complex effect on thalamocortical circuits after dopamine loss. Our results support the hypothesis that parvalbumin interneurons in the motor cortex are implicated in integrating local circuits, particularly in recurrent and feedback inhibition (Nashef et al., 2021), and are likely to be involved in shaping responses to thalamic input, similar to their roles in ventral medial thalamus projections to the anterolateral motor cortex (Guo et al., 2018).

Collectively, these results emphasize that different groups of M1 neural spiking activities are likely to be a contributing circuit feature for the generation of aberrant synchronization with complexes and patterns in individuals with PD (McColgan et al., 2020; Underwood and Parr-Brownlie, 2021) and highlight a changed functional coupling between the PF and the motor cortex. A key neural mechanism may involve the M1 as a major source of driving excitation to regulate the thalamus and in or between different nodes of thalamocortical circuits (McColgan et al., 2020). This cortical-mediated synchronization mechanism may produce the large-scale integration of information across thalamocortical circuits (Sherman, 2017). Therefore, our data argue for extending the influential concept of pathological variation in thalamocortical pathways from firing rates to the ongoing firing-correlated rhythmic band of LFPs following chronic dopamine depletion.

### Anatomical Reciprocal Connectivity Between the Parafascicular Nucleus and the M1

Accumulating evidence from our electrophysiological recordings highlights that the reciprocal action between PF and M1 neurons in parkinsonism rats is involved in food catching movements. Next, our data provide anatomical evidence for the existence of afferent and efferent bidirectional reciprocal connectivity pathways between the PF and the M1, which were detected by neuroanatomical tracing with anterograde and retrograde fluorescent tracer viruses to identify PF-M1 and M1-PF projection neurons, respectively. This experiment suggests significant connectivity with bidirectional, rather than unidirectional, flow of information in the PF and M1. Unlike other typical thalamic nuclei, the main projection of the PF has the highest density of striatum projections among subcortical structures and only minor anatomical projections to and from the cerebral cortex (Unzai et al., 2017). The findings indicate that the functional properties of the PF are dependent not only on their reciprocal connection with the striatum but also on their afferent and efferent connections with the M1. This outcome is consistent with previous studies showing that thalamic nuclei reciprocally connect with the cerebral cortex by receiving input from and projecting to the cortical region (Sherman, 2016; Unzai et al., 2017; Mandelbaum et al., 2019). The reciprocal relationship between the thalamus and the cerebral cortex is presumed to play a key role in physiological and pathological motor disturbances (Brazhnik et al., 2016; Guo et al., 2017; Reis et al., 2019; Gaidica et al., 2020). The complex connectivity between the PF and the M1 is likely important for processes related to movement information to be efficiently transformed to final motor commands. Nevertheless, an illustration of the reciprocal connectivity between the PF and the M1 only using anatomical methods is insufficient. Therefore, a limitation of this study is the lack of evaluation by optogenetic or electrical stimulation to confirm the functional connectivity between the PF and the M1.

## Conclusion

The evidence from the literature and the present results reveals a complicated relationship of neuronal activity in animal PD models, which from different subpopulations of PF and M1 neuron in thalamocortical circuits. Our data place a special emphasis on confirming a bidirectional pattern of communication in anatomy and a reciprocal pattern across spikes with LFP frequency bands and their differential changes in response to chronic dopamine depletion. The results support the hypothesis that no unique defects, such as excessive beta frequency synchrony, exist that might be responsible for PD pathological mechanisms; instead, other rhythms and multiple alterations in regional anatomical and functional synchronization in thalamocortical circuits potentially contribute to the pathological complexity of PD. Overall, our study provides empirical evidence for a circuit-level mechanism of PD with a special emphasis on reciprocal interactions between the PF and the M1.

## Data Availability Statement

The original contributions presented in the study are included in the article/Supplementary Material; further inquiries can be directed to the corresponding author/s.

## Ethics Statement

The animal study was reviewed and approved by the Shandong Normal University Ethics Review Board.

## Author Contributions

ML contributed to formal analysis, performing experiments, data analysis, and writing of the original draft. XZ contributed to formal analysis, data analysis, methodology, and software. QH and SS contributed to data curation, methodology, and software. DC contributed to data curation, validation, and software. FC and XW contributed to data curation, validation, and resources. YS, YL, ZZ, HF, XS, and XY contributed to software and data analysis. HS contributed to data curation, resources, supervision, validation and funding acquisition. MW contributed to conceptualization, resources, supervision, funding acquisition, project administration, and writing, reviewing, and editing the manuscript. All authors contributed to the article and approved the submitted version.

## Conflict of Interest

The authors declare that the research was conducted in the absence of any commercial or financial relationships that could be construed as a potential conflict of interest.

## Publisher’s Note

All claims expressed in this article are solely those of the authors and do not necessarily represent those of their affiliated organizations, or those of the publisher, the editors and the reviewers. Any product that may be evaluated in this article, or claim that may be made by its manufacturer, is not guaranteed or endorsed by the publisher.

## Footnotes

1
http://www.plexon.com

2
http://chronux.org

## Supplementary Material

Supplementary Figure 1 GAD immunoreactivity in the PF. Coronal section of the PF illustrating immunoreactivity of NeuN (green) and GAD67 (red) positive neurons **(A)**. Scale bar = 75 μm. The framed area in A is magnified in **(B)**. Scale bar = 25 μm.

## Abbreviations

6-OHDA: 6-hydroxydopamine
BS: broad spike
GABA: γ-aminobutyric acidergic
GLU: glutamatergic
ISIs: inter-spike interval histograms
LFP: local field potential
M1: primary motor cortex
MFB: medial forebrain bundle
MWUT: Mann–Whitney *U*-test
NS: narrow spike
PV: parvalbumin
PBS: phosphate buffer solution
PD: Parkinson’s disease
PETH: perievent time histograms
PF: parafascicular nucleus
TH: tyrosine hydroxylase
DA: dopamine.
