# Supplementary figures and images for: Functional Interactions Between the Parafascicular Thalamic Nucleus and Motor Cortex Are Altered in Hemiparkinsonian Rat
Source: Front Aging Neurosci. 2022 May 23;14:800159. doi: 10.3389/fnagi.2022.800159 (PMC9168077; doi:10.3389/fnagi.2022.800159)

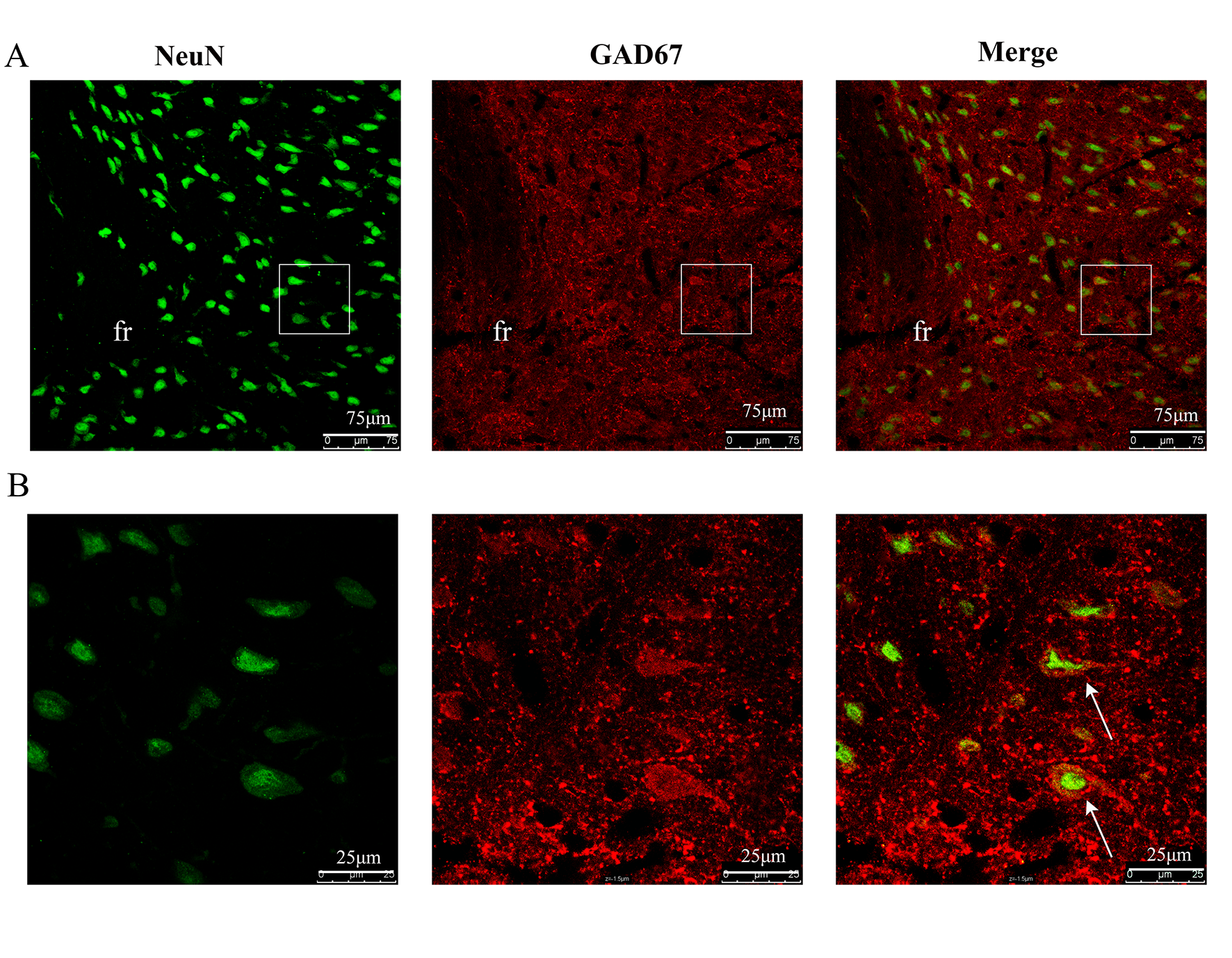

Supplement: Supplementary Figure 1 — GAD immunoreactivity in the PF. Coronal section of the PF illustrating immunoreactivity of NeuN (green) and GAD67 (red) positive neurons (A). Scale bar = 75 μm. The framed area in A is magnified in (B). Scale bar = 25 μm. [file Image_1.tif]
